# Supplementary material for: Isatin Bis-Imidathiazole Hybrids Identified as FtsZ Inhibitors with On-Target Activity Against Staphylococcus aureus
Source: Antibiotics (Basel). 2024 Oct 19;13(10):992. doi: 10.3390/antibiotics13100992 (PMC11505029; doi:10.3390/antibiotics13100992)
Supplement: Supplementary file 1 [file antibiotics-13-00992-s001.zip › antibiotics-3244517-supplementary.pdf]

Article

Isatin bis-imidathiazole hybrids identified as FtsZ inhibitors with on-target activity against *Staphylococcus aureus*

Rita Morigi [1], Daniele Esposito [1], Matteo Calvaresi [2,3], Tainah Dorina Marforio [2,3], Giovanna Angela Gentilomi [4,5], Francesca Bonvicini [4,*] and Alessandra Locatelli [1]

[1] Department of Pharmacy and Biotechnology, Alma Mater Studiorum-University of Bologna, Via Belmeloro 6, 40126 Bologna, Italy; rita.morigi@unibo.it (R.M.); daniele.esposito6@unibo.it (D.E.); alessandra.locatelli@unibo.it (A.L.)

[2] Department of Chemistry "Giacomo Ciamician", Alma Mater Studiorum-University of Bologna, Via Selmi 2, Bologna, 40126, Italy; matteo.calvaresi3@unibo.it (M.C.); tainah.marforio2@unibo.it (T.M.)

[3] IRCCS Azienda Ospedaliero-Universitaria di Bologna, Bologna, Italy,matteo.calvaresi3@unibo.it (M.C.); tainah.marforio2@unibo.it (T.M.)

[4] Department of Pharmacy and Biotechnology, Alma Mater Studiorum-University of Bologna, Via Massarenti 9, 40138 Bologna, Italy; giovanna.gentilomi@unibo.it (G.G.)

[5] Division of Microbiology, IRCCS Azienda Ospedaliero-Universitaria di Bologna, Via Massarenti 9, 40138 Bologna, Italy; giovanna.gentilomi@unibo.it (G.G.)

* Correspondence: francesca.bonvicini4@unibo.it (F.B.)

Contents:

Figure S1: ^{1}H NMR and ^{13}C NMR spectra pag. 3-20

Figure S2: FtsZ multiple alignment and superposition of 3D structures pag. 21

Table S1: Percentage values of the hemolytic activity of derivative **11** on hRBCs pag. 22

Table S2: Antibiotic-resistance profile of the clinical isolates of S. *aureus* pag. 22

Figure S3. Post-processing analysis of MD trajectory of derivative **11** in the interdomain cleft pag. 23

Table S3. Inter-molecular hydrogen bond analysis of MD trajectory of derivative **11** in the interdomain cleft pag. 24

Figure S4. Post-processing analysis of MD trajectory of derivative **11** in the GTP-binding pocket pag. 25

Table S4. Inter-molecular hydrogen bond analysis of MD trajectory of derivative **11** in the GTP-binding pocket pag. 26

Figure S5. Post-processing analysis of MD trajectory of apo-FtsZ dimer pag. 27

Table S5. Intra-molecular (monomer 1) hydrogen bond analysis of MD trajectory of apo-FtsZ dimer pag. 27-29

Table S6. Intra-molecular (monomer 2) hydrogen bond analysis of MD trajectory of

apo-FtsZ dimer pag. 29-31

Table S7. Inter-molecular (monomer 1-2) hydrogen bond analysis of MD trajectory of apo-FtsZ dimer pag. 31

Figure S6. Post-processing analysis of MD trajectory of FtsZ dimer in presence of GTP and Ca^{2+} pag. 32

Table S8. Intra-molecular (monomer 1) hydrogen bond analysis of MD trajectory of FtsZ dimer in presence of GTP and Ca^{2+} pag. 32-34

Table S9. Intra-molecular (monomer 2) hydrogen bond analysis of MD trajectory of FtsZ dimer in presence of GTP and Ca^{2+} pag. 34-36

Table S10. Inter-molecular (monomer 1-2) hydrogen bond analysis of MD trajectory of FtsZ dimer in presence of GTP and Ca^{2+} pag. 36

Figure S7. Post-processing analysis of MD trajectory of FtsZ dimer in presence of GTP, Ca^{2+} and compound **11** pag. 37

Table S11. Intra-molecular (monomer 1) hydrogen bond analysis of MD trajectory of FtsZ dimer in presence of GTP, Ca^{2+} and compound **11** pag. 37-39

Table S12. Intra-molecular (monomer 2) hydrogen bond analysis of MD trajectory of FtsZ dimer in presence of GTP, Ca^{2+} and compound **11** pag. 39-41

Table S13. Inter-molecular (monomer 1-2) hydrogen bond analysis of MD trajectory of FtsZ dimer in presence of GTP, Ca^{2+} and compound **11** pag. 41

Figure S1: ^{1}H and ^{13}C NMR spectra of the new compounds.

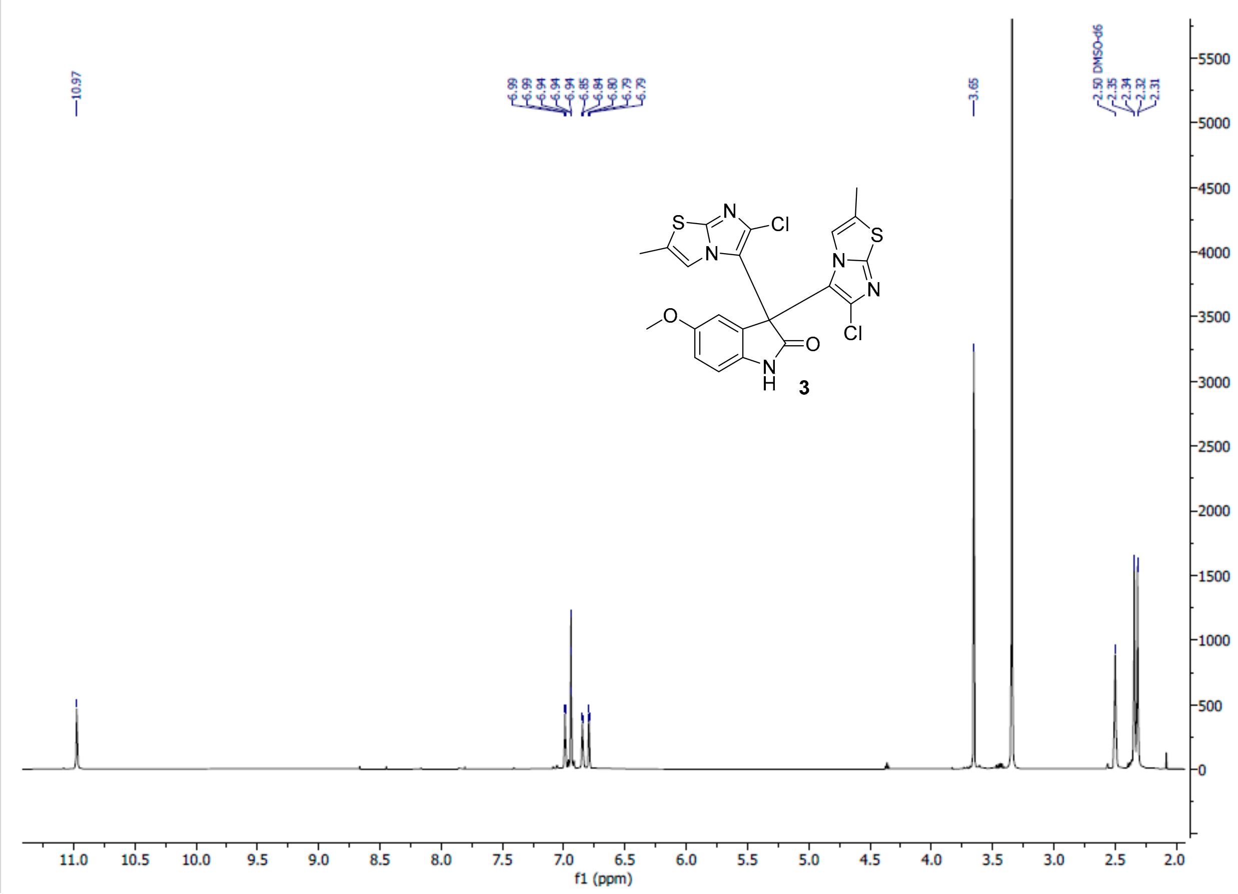

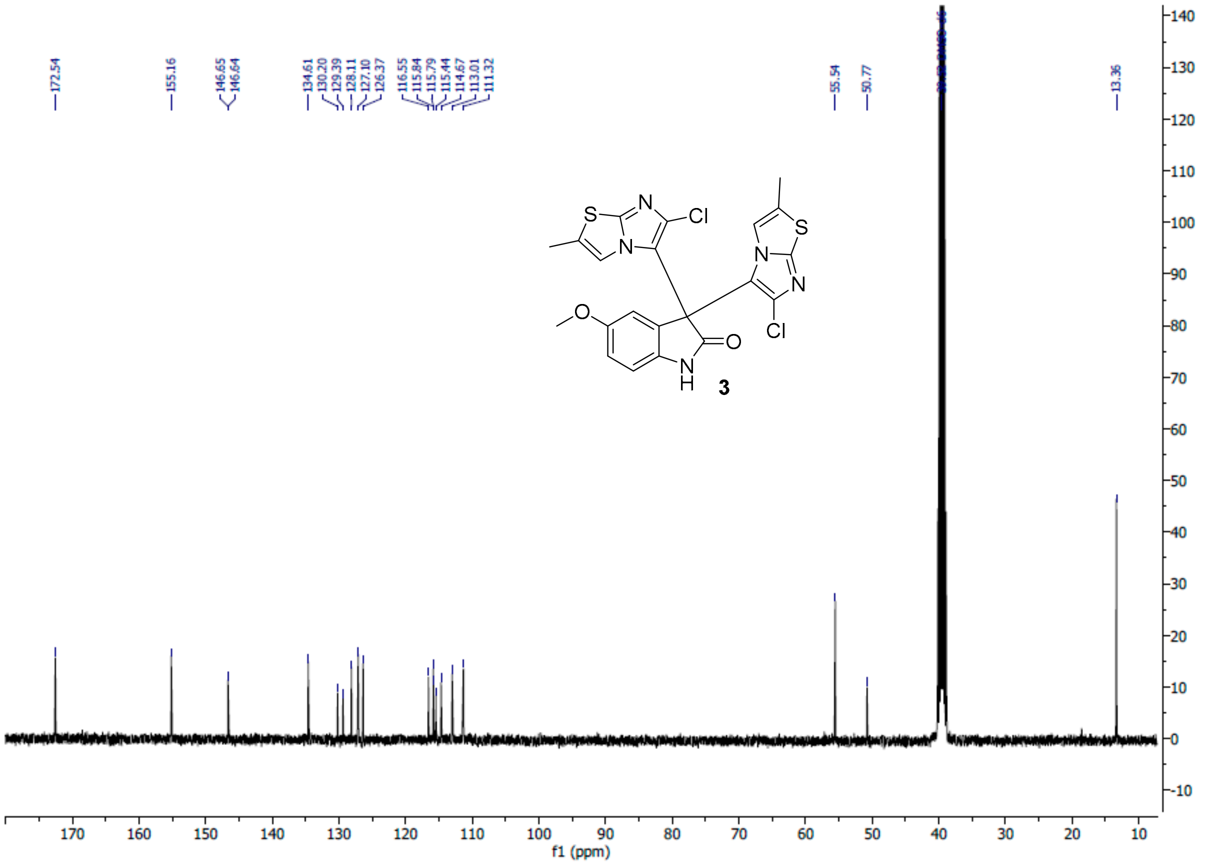

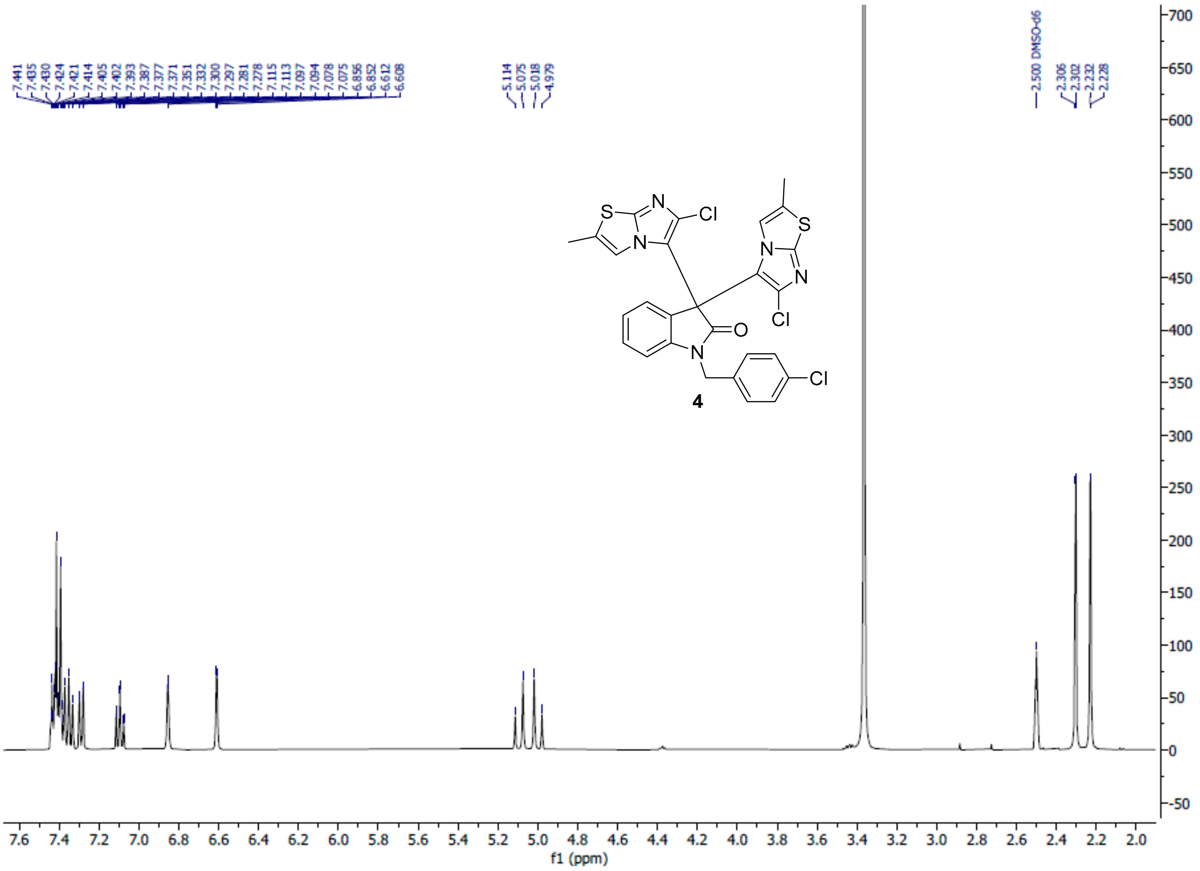
7.441
7.435
7.430
7.424
7.421
7.414
7.405
7.402
7.393
7.387
7.377
7.371
7.351
7.332
7.300
7.297
7.281
7.278
7.115
7.113
7.097
7.094
7.078
7.075
6.856
6.852
6.612
6.608
5.114
5.075
5.018
4.979
2.500 DMSO-d6
2.306
2.302
2.232
2.228
Cl
S
N
4
f1 (ppm)

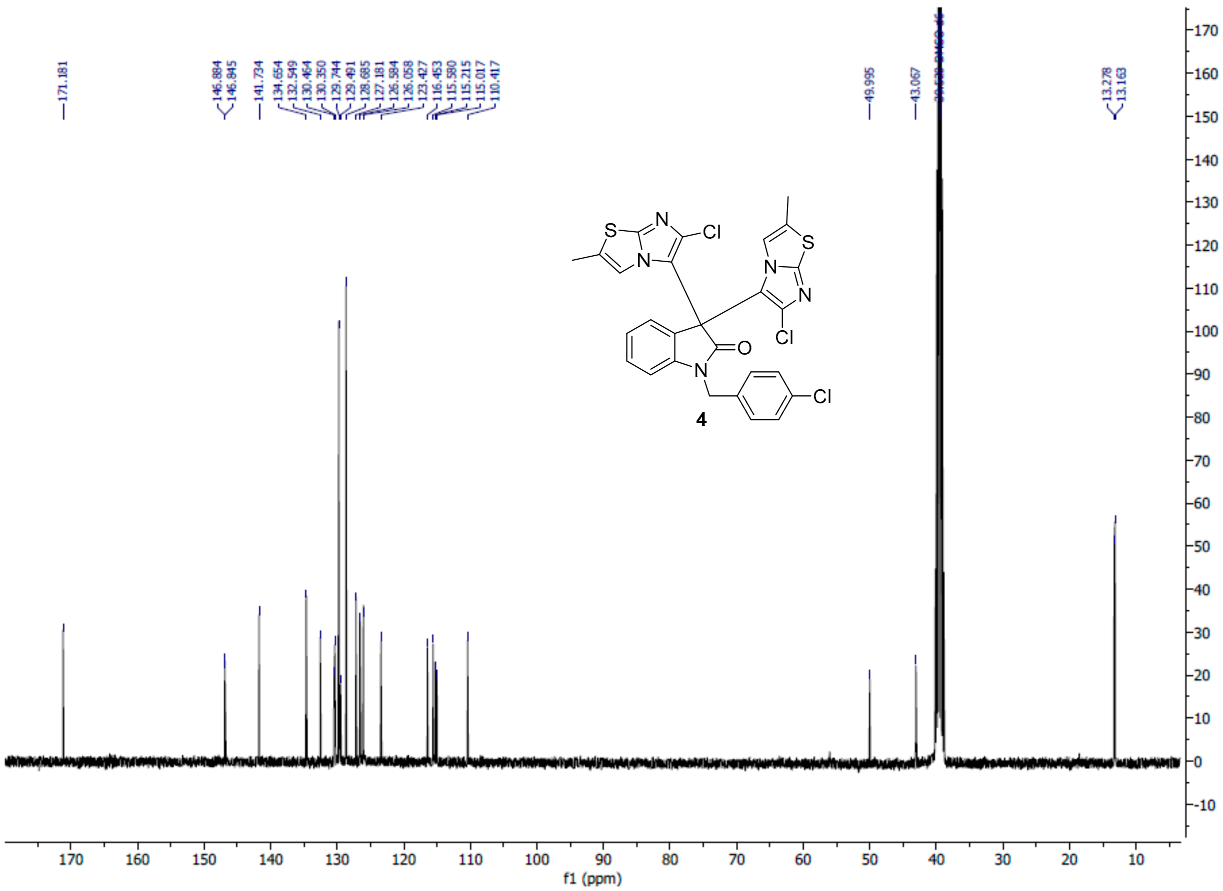
171.181
146.884
146.845
141.734
134.654
132.549
130.464
130.350
129.744
129.491
128.685
127.181
126.584
126.058
123.427
116.453
115.580
115.215
115.017
110.417
49.995
43.067
DMSO-d6
13.278
13.163
Cl
S
N
4
f1 (ppm)

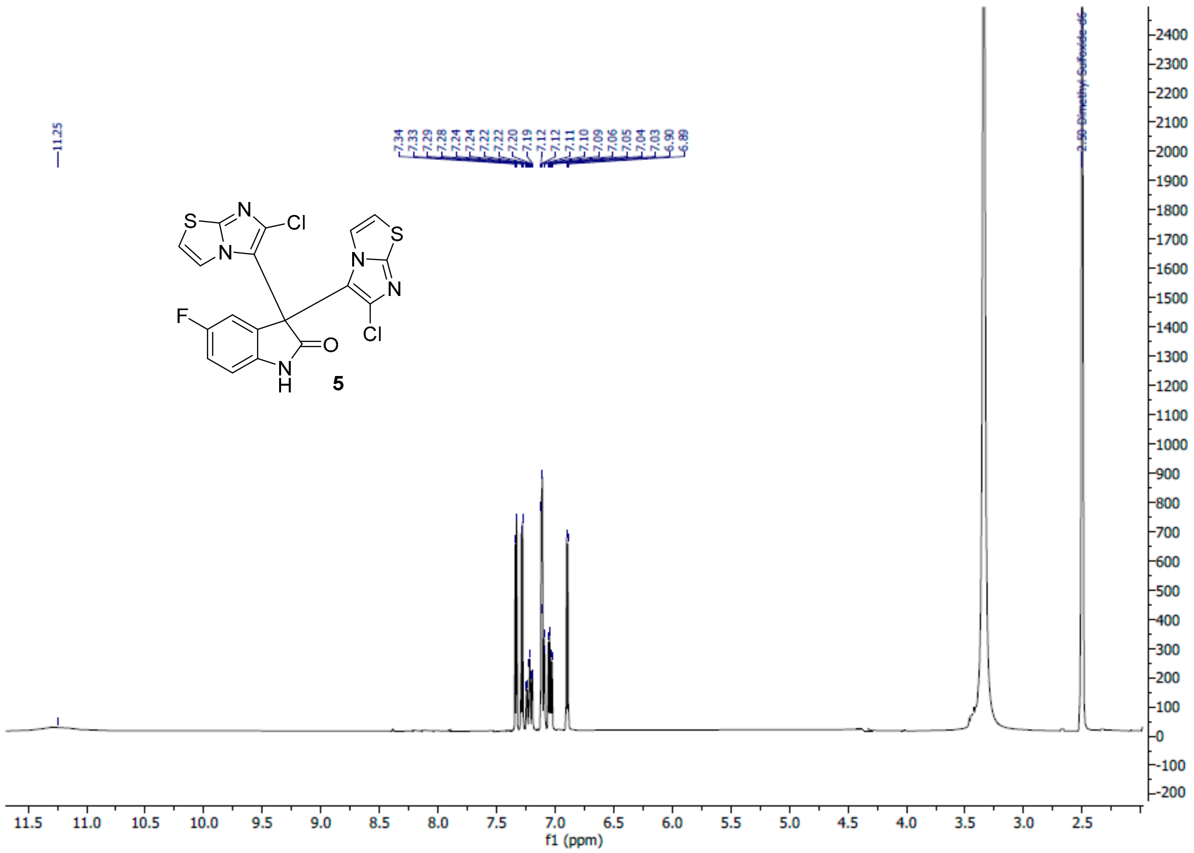
11.25
7.34 7.33 7.29 7.28 7.24 7.24 7.22 7.22 7.20 7.19 7.12 7.12 7.11 7.10 7.09 7.06 7.05 7.04 7.03 6.90 6.89
2.50 Dimethyl Sulfoxide-d6
S N Cl
S N
F
Cl
O
N H
5
f1 (ppm)

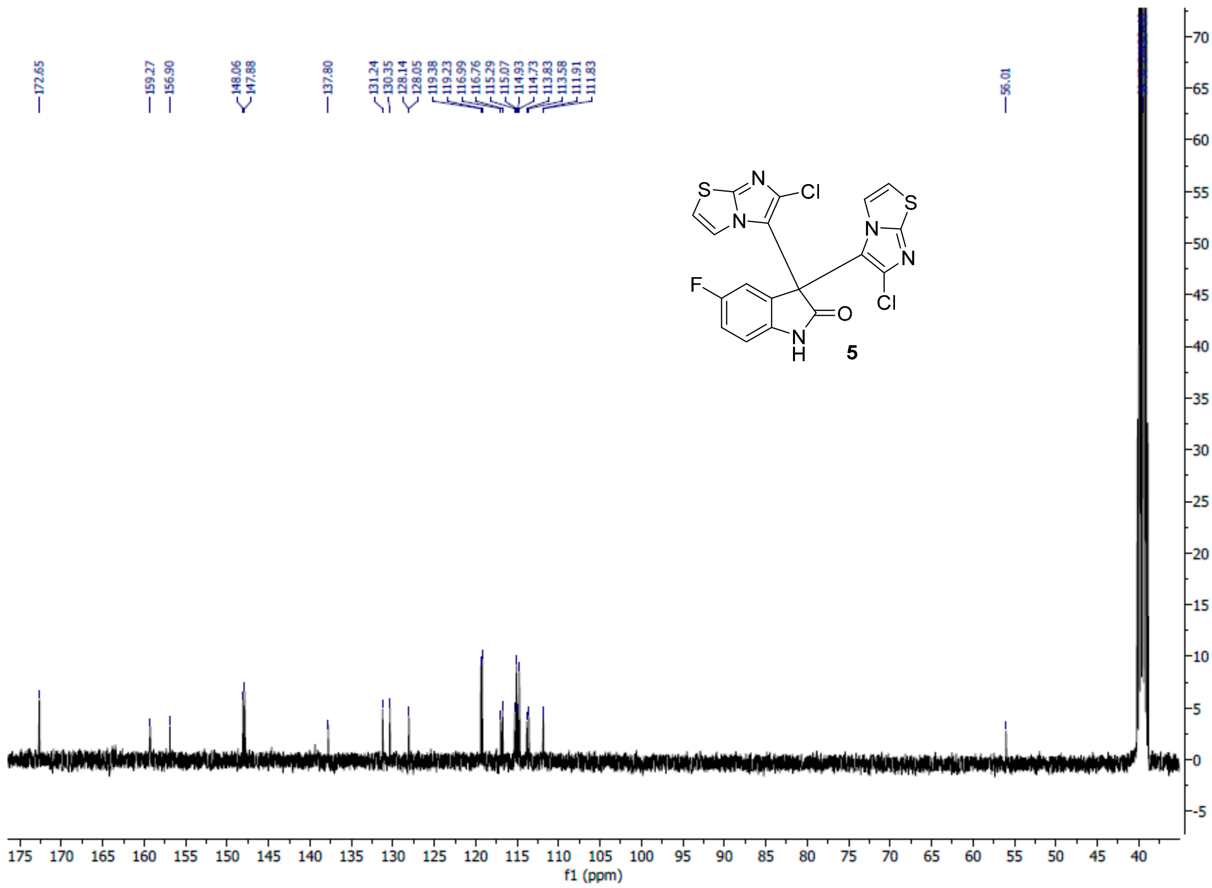
172.65
159.27 156.90
148.06 147.88
137.80
131.24 130.35 128.14 128.05 119.38 119.23 116.99 116.76 115.29 115.07 114.93 114.73 113.83 113.58 111.91 111.83
56.01
S N Cl
S N
F
Cl
O
N H
5
f1 (ppm)

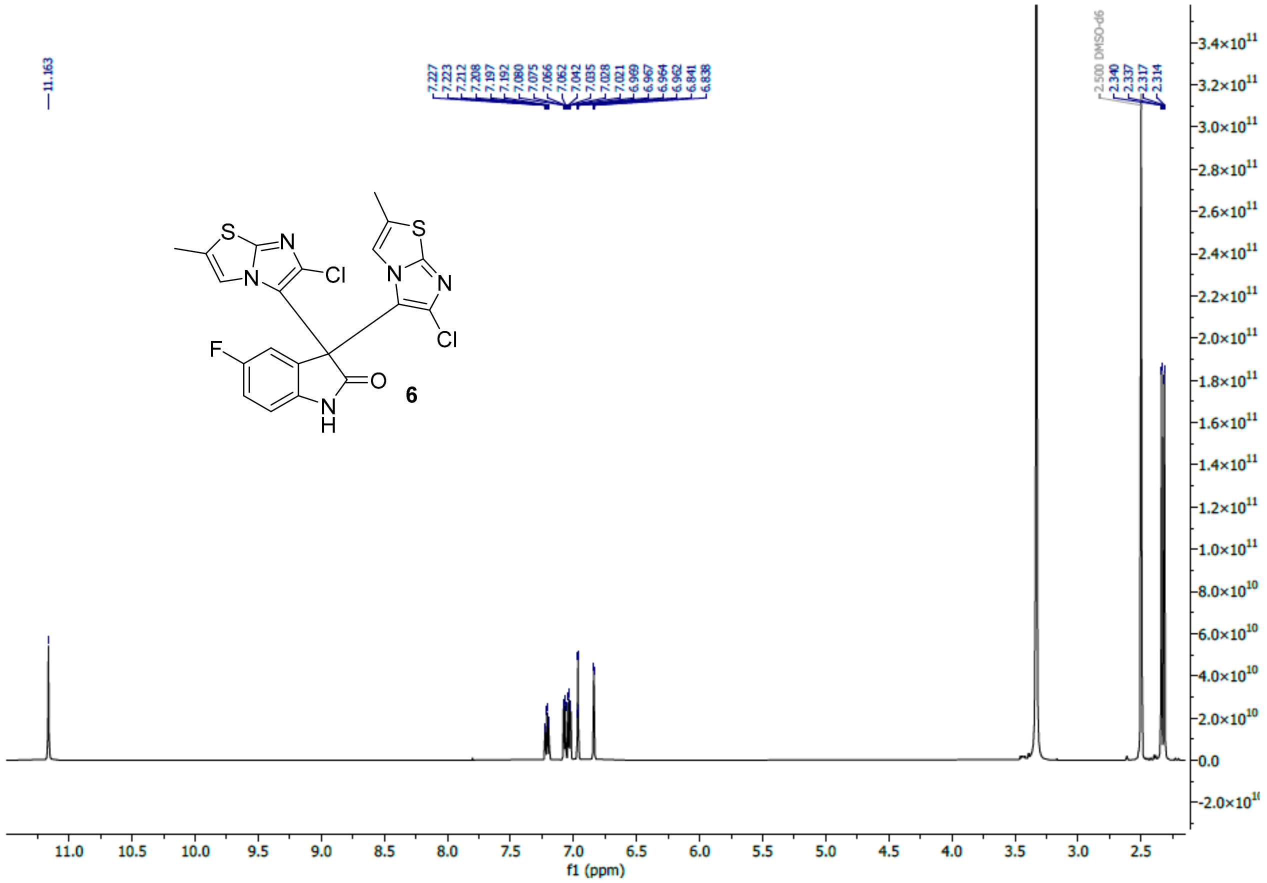
11.163
7.227 7.223 7.212 7.208 7.197 7.192 7.080 7.075 7.066 7.062 7.042 7.035 7.028 7.021 6.969 6.967 6.964 6.962 6.841 6.838
2.500 DMSO-d6
2.340 2.337 2.317 2.314
S
N
Cl
F
O
N
H
6
f1 (ppm)

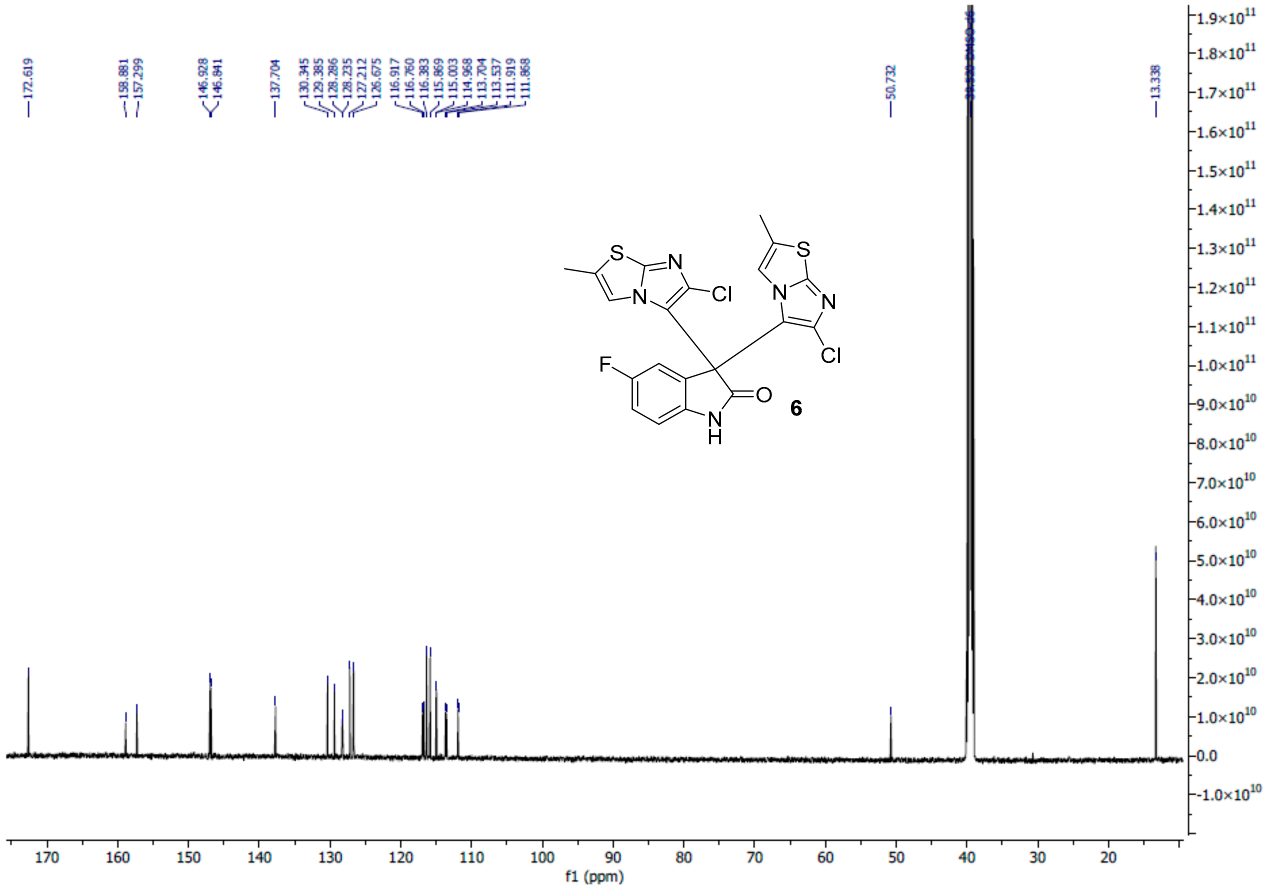
172.619
158.881 157.299
146.928 146.841
137.704
130.345 129.385 128.286 128.235 127.212 126.675
116.917 116.760 116.383 115.869 115.003 114.968 113.704 113.537 111.919 111.868
50.732
13.338
S
N
Cl
F
O
N
H
6
f1 (ppm)

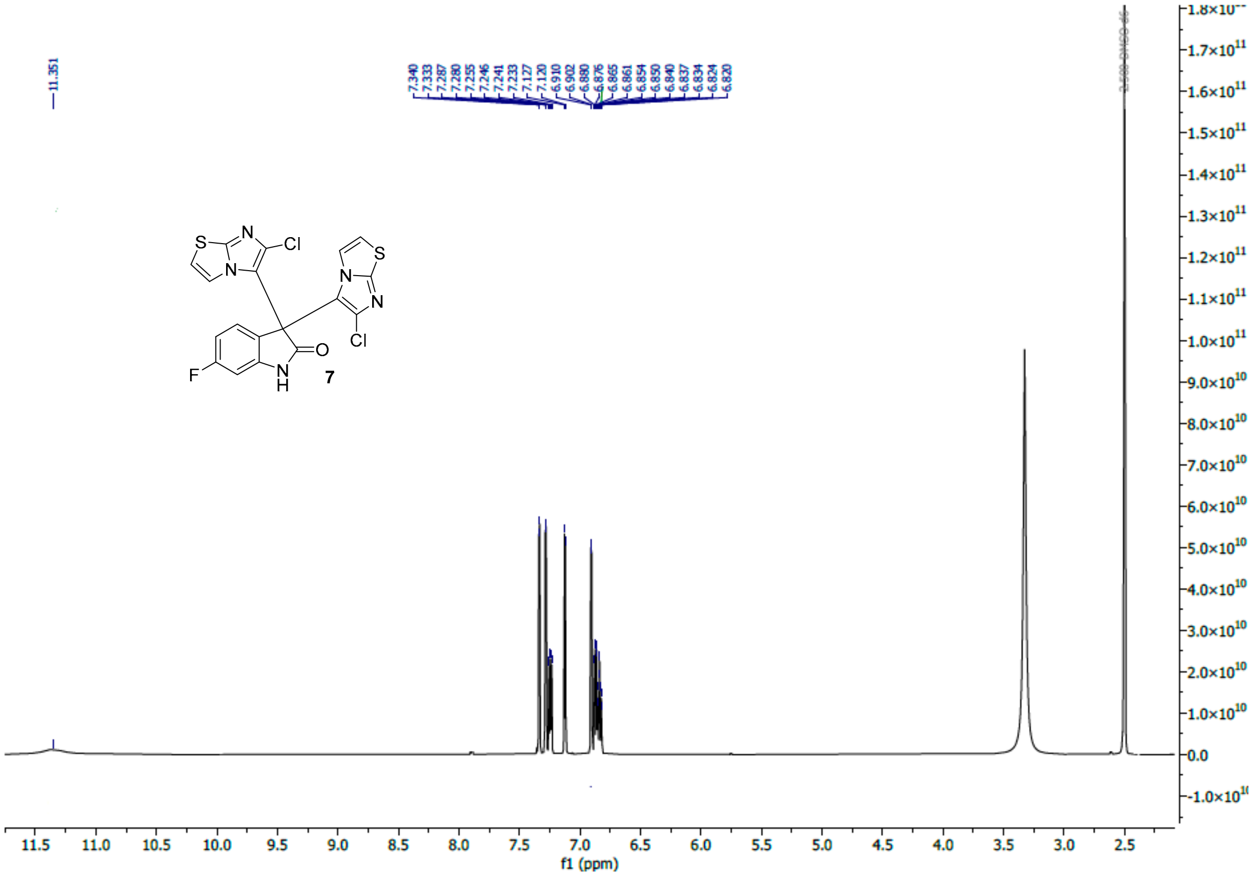
11.351
7.340
7.333
7.287
7.280
7.255
7.246
7.241
7.233
7.127
7.120
6.910
6.902
6.880
6.876
6.865
6.861
6.854
6.850
6.840
6.837
6.834
6.824
6.820
S
N
Cl
N
S
N
N
Cl
F
N
H
O
7
f1 (ppm)

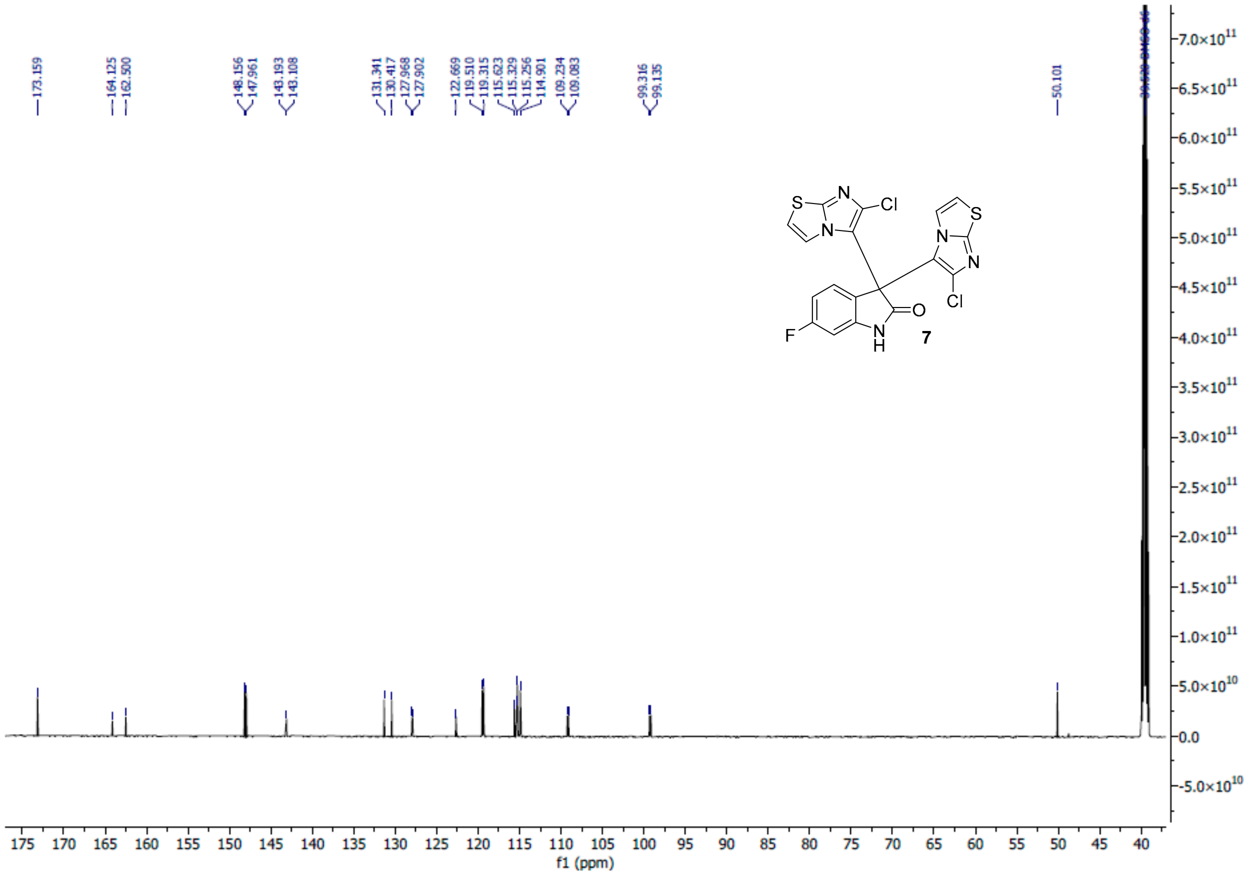
173.159
164.125
162.500
148.156
147.961
143.193
143.108
131.341
130.417
127.968
127.902
122.669
119.510
119.315
115.623
115.329
115.256
114.901
109.234
109.083
99.316
99.135
50.101
S
N
Cl
N
S
N
N
Cl
F
N
H
O
7
f1 (ppm)

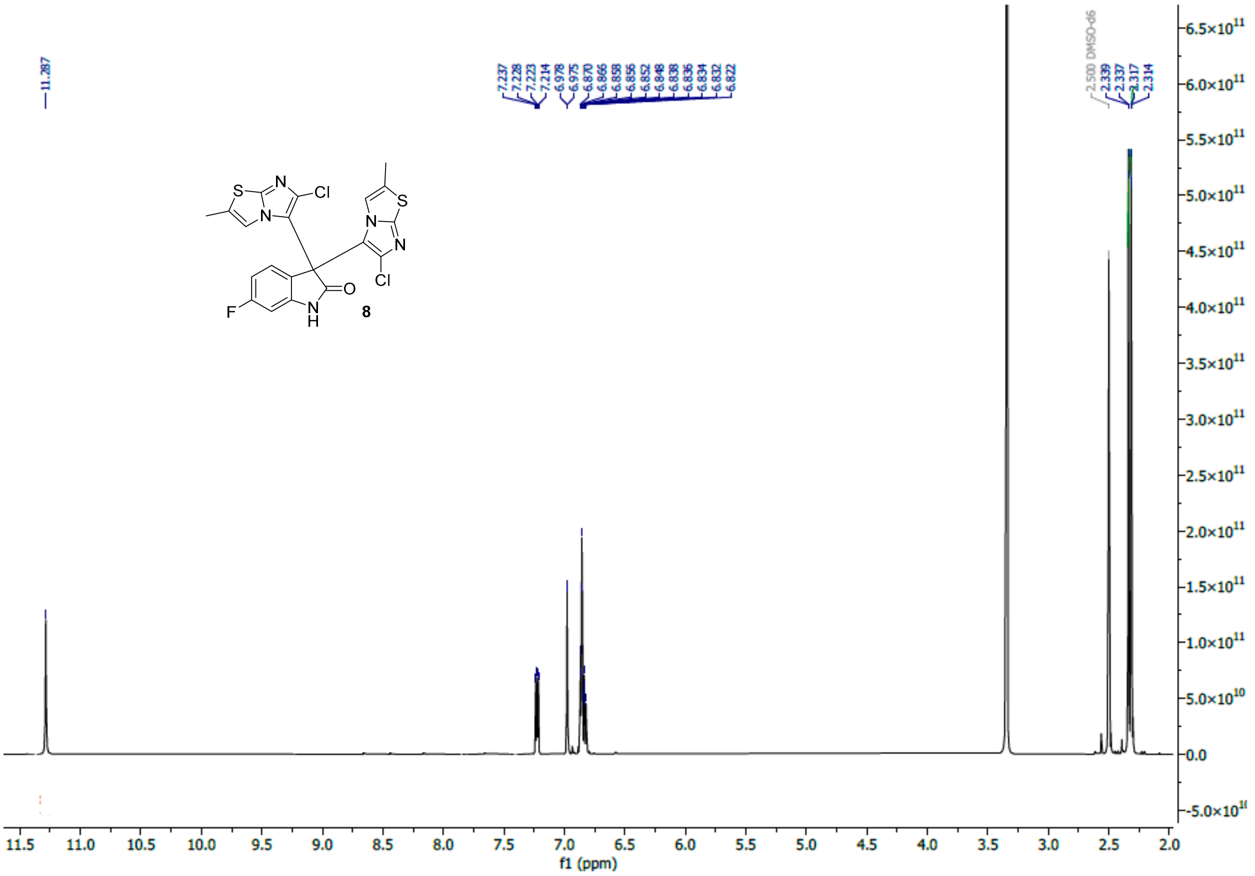
11.287
7.237
7.228
7.223
7.214
6.978
6.975
6.870
6.866
6.858
6.856
6.852
6.848
6.838
6.836
6.834
6.832
6.822
2.500 DMSO-d6
2.339
2.337
2.317
2.314
S
N
Cl
F
NH
O
8
f1 (ppm)

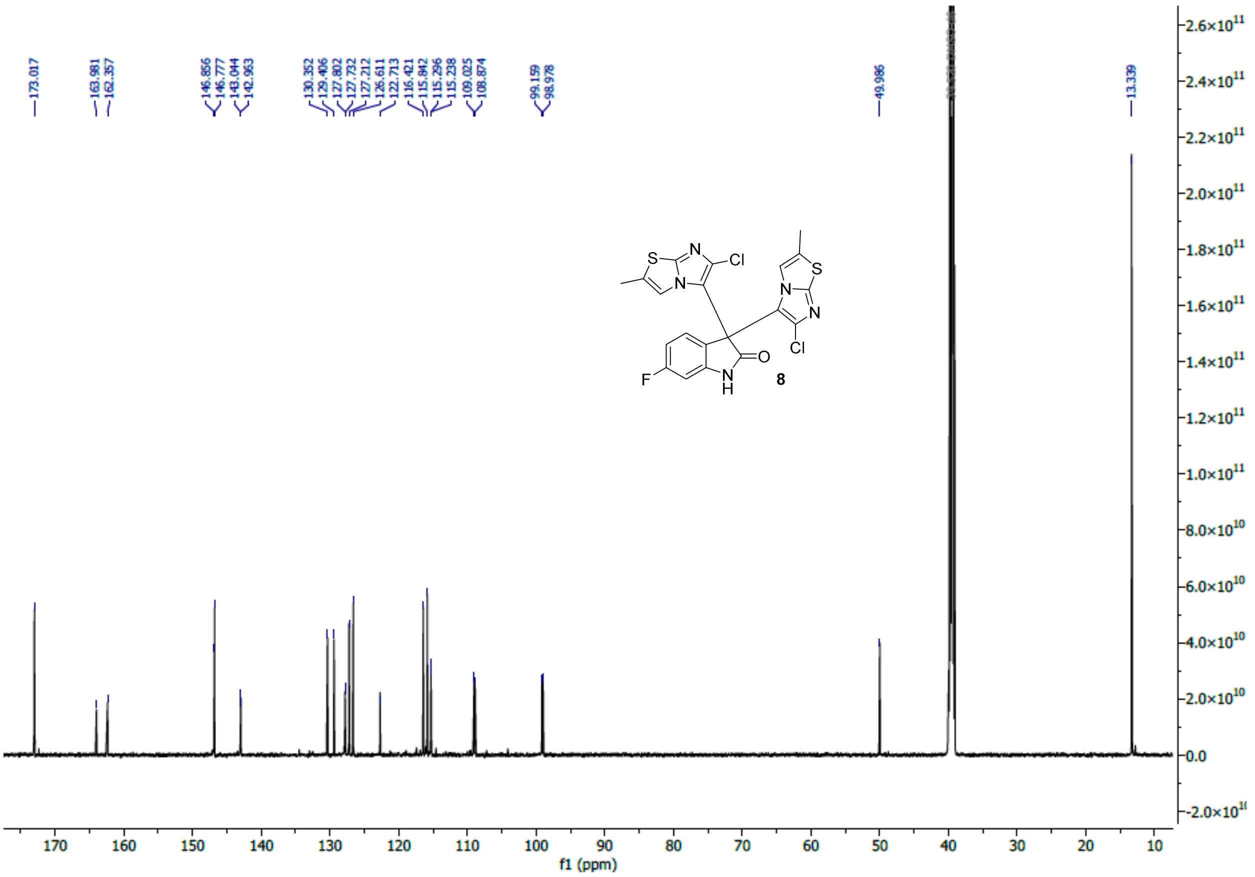
173.017
163.981
162.357
146.856
146.777
143.044
142.963
130.352
129.406
127.802
127.732
127.212
126.611
122.713
116.421
115.842
115.296
115.238
109.025
108.874
99.159
98.978
49.986
13.339
S
N
Cl
F
NH
O
8
f1 (ppm)

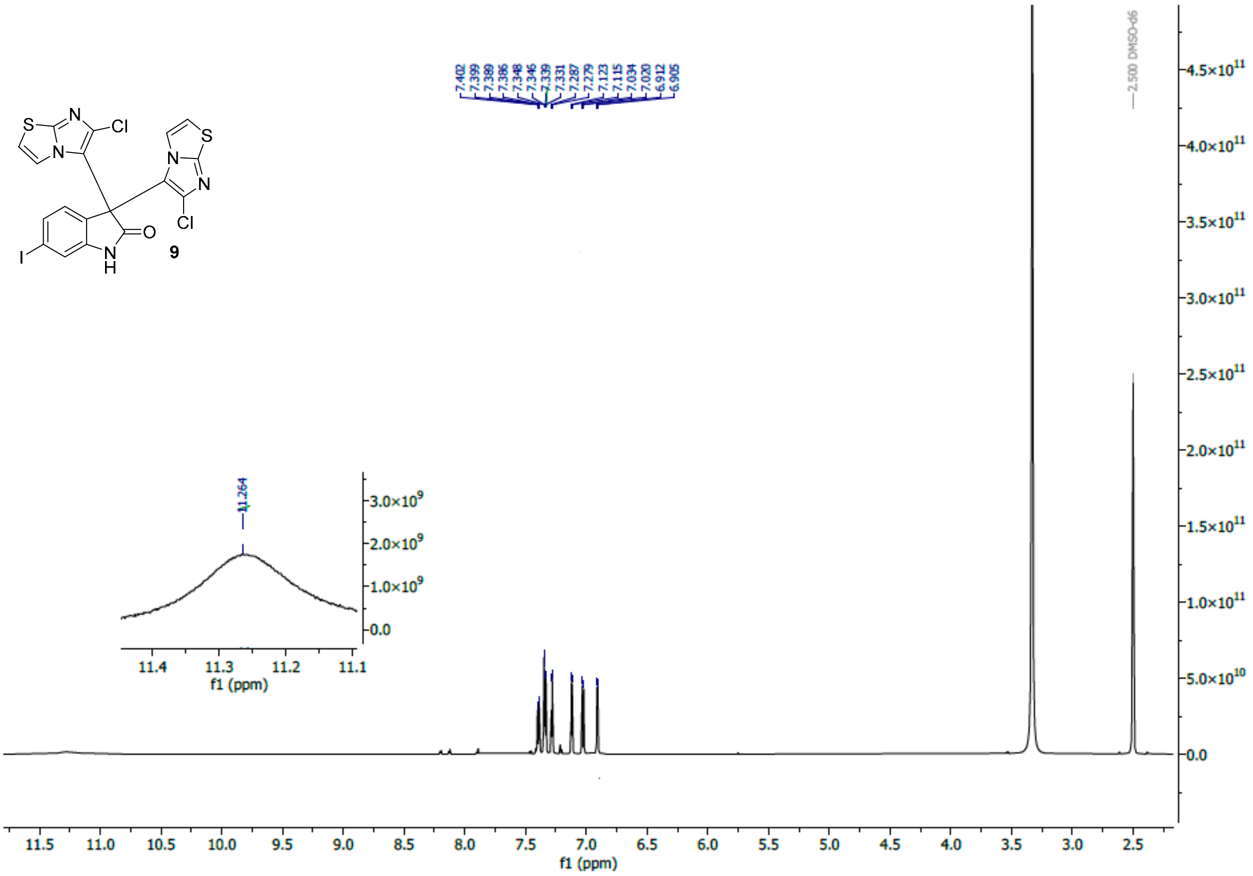
7.402 7.399 7.389 7.386 7.348 7.346 7.339 7.331 7.287 7.279 7.123 7.115 7.034 7.020 6.912 6.905
2.500 DMSO-d6
11.264
9
f1 (ppm)

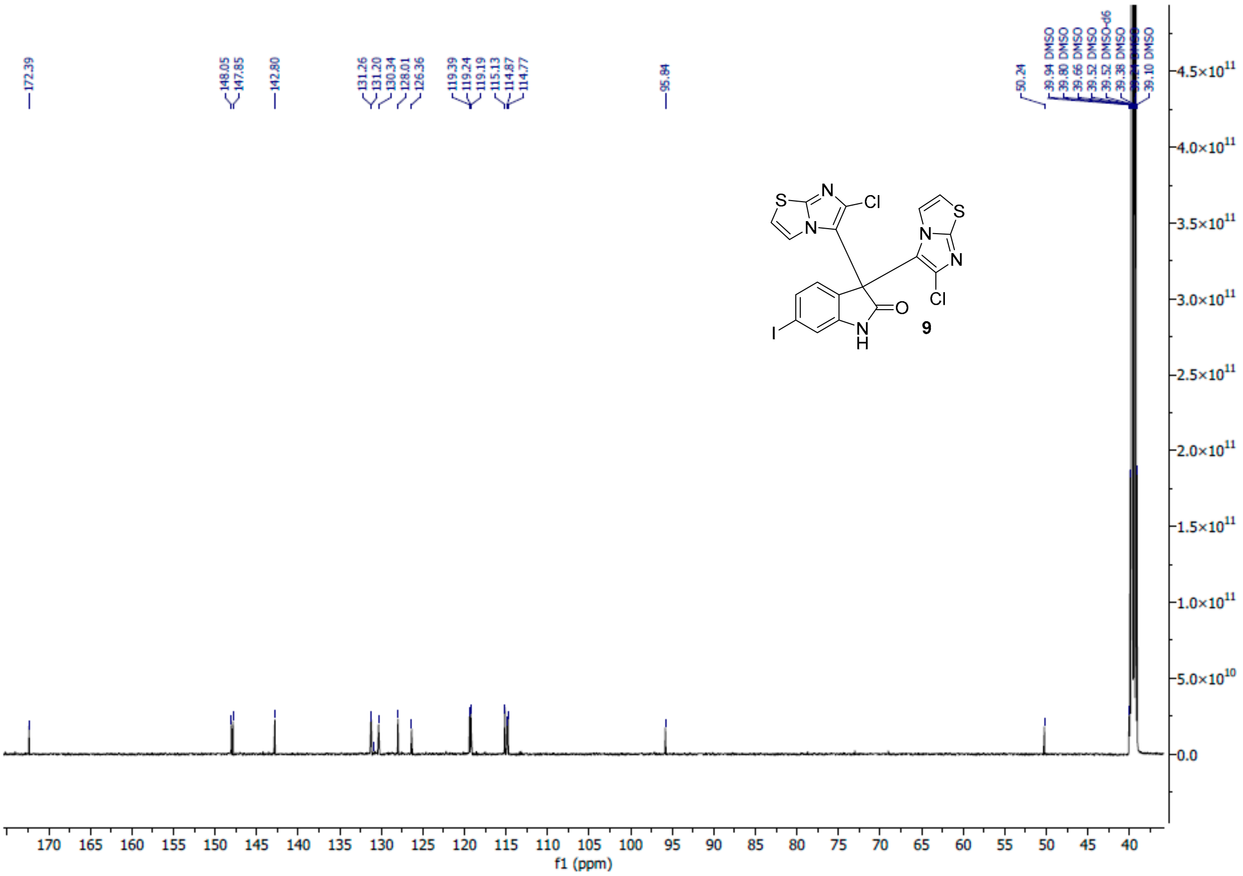
172.39
148.05 147.85
142.80
131.26 131.20 130.34 128.01 126.36
119.39 119.24 119.19 115.13 114.87 114.77
95.84
50.24
39.94 DMSO 39.80 DMSO 39.66 DMSO 39.52 DMSO-d6 39.38 DMSO 39.24 DMSO 39.10 DMSO
9
f1 (ppm)

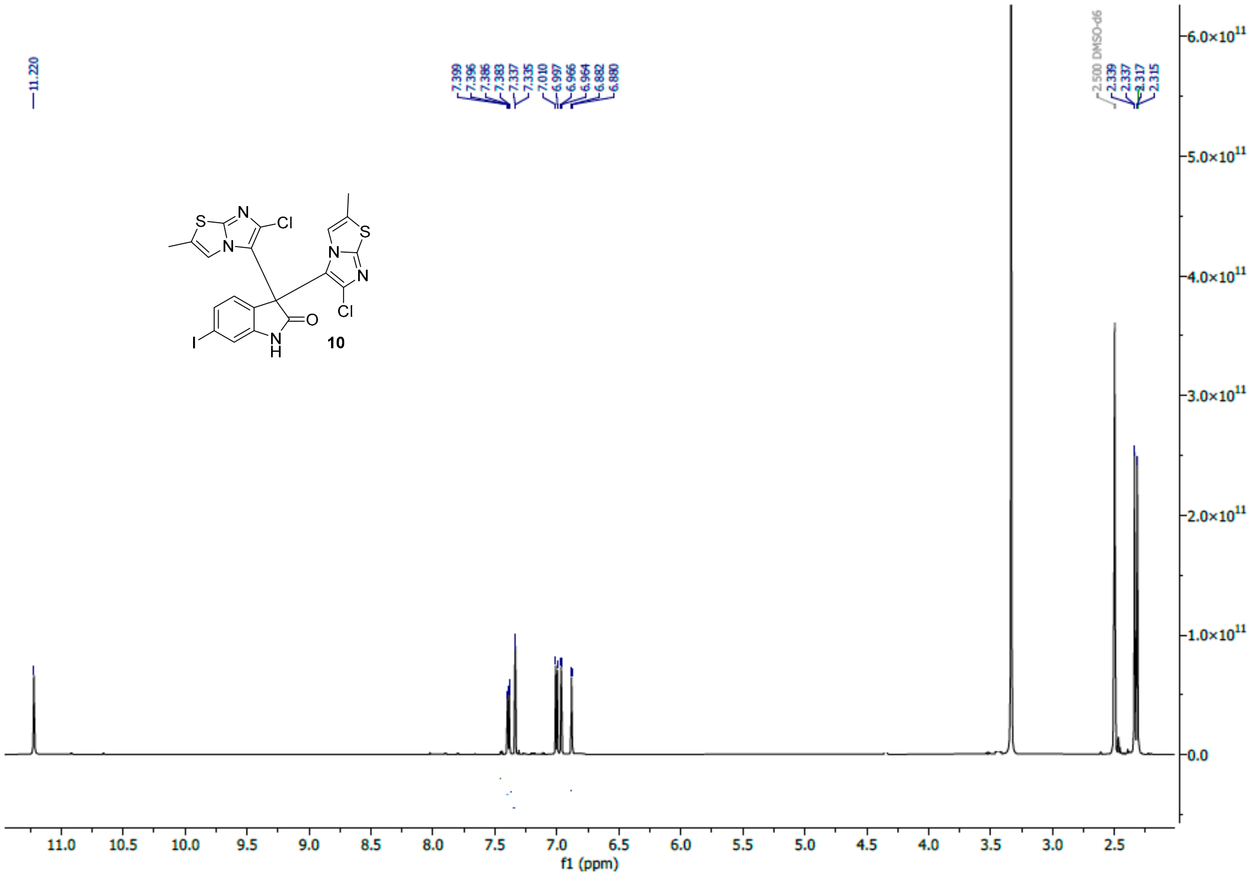
11.220
7.399
7.396
7.386
7.383
7.337
7.335
7.010
6.997
6.966
6.964
6.882
6.880
2.500 DMSO-d6
2.339
2.337
2.317
2.315
S
N
Cl
N
S
N
N
Cl
O
I
N
H
10
f1 (ppm)

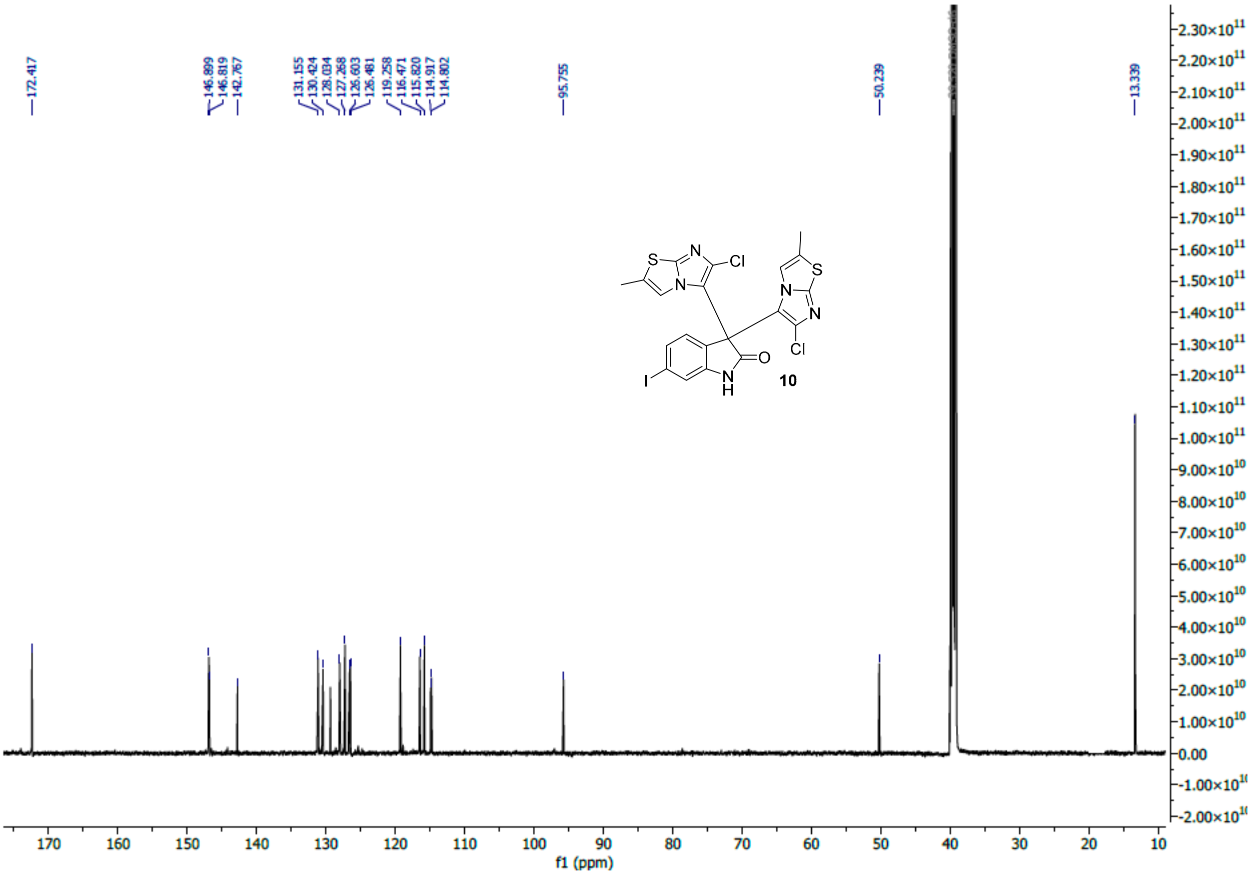
172.417
146.899
146.819
142.767
131.155
130.424
128.034
127.268
126.603
126.481
119.258
116.471
115.820
114.917
114.802
95.755
50.239
13.339
S
N
Cl
N
S
N
N
Cl
O
I
N
H
10
f1 (ppm)

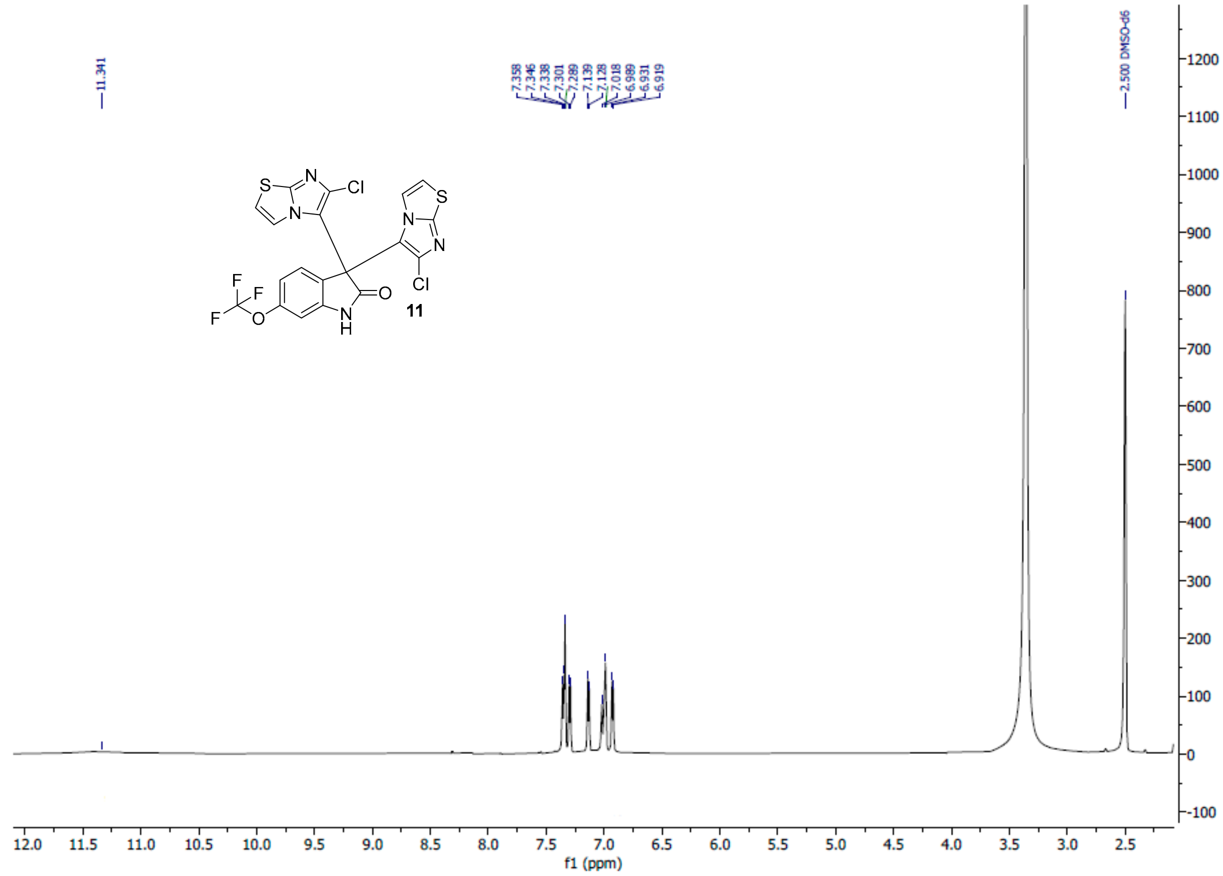
11.341
7.358
7.346
7.338
7.301
7.289
7.139
7.128
7.018
6.989
6.931
6.919
2.500 DMSO-d6
S
N
Cl
N
Cl
F
O
NH
O
11
f1 (ppm)

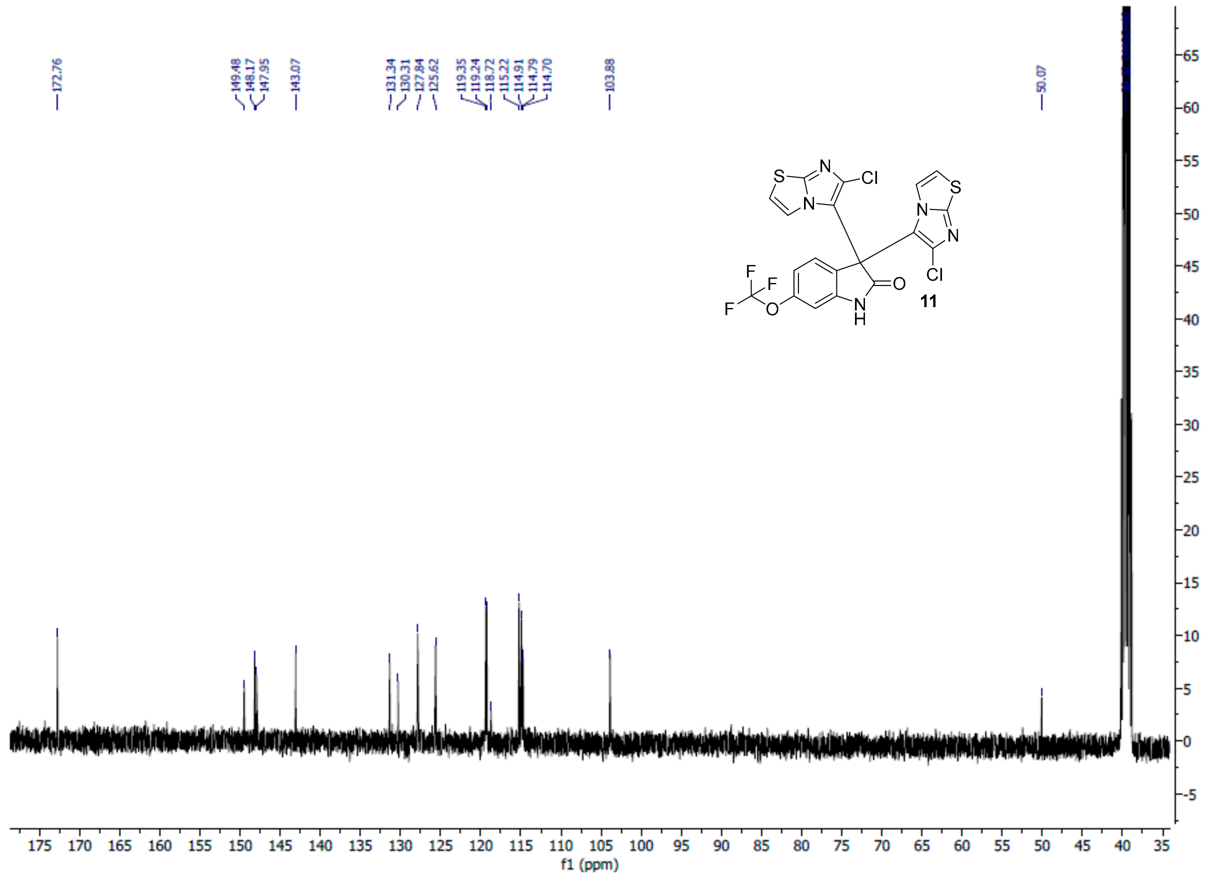
172.76
149.48
148.17
147.95
143.07
131.34
130.31
127.84
125.62
119.35
119.24
118.72
115.22
114.91
114.79
114.70
103.88
50.07
S
N
Cl
N
Cl
F
O
NH
O
11
f1 (ppm)

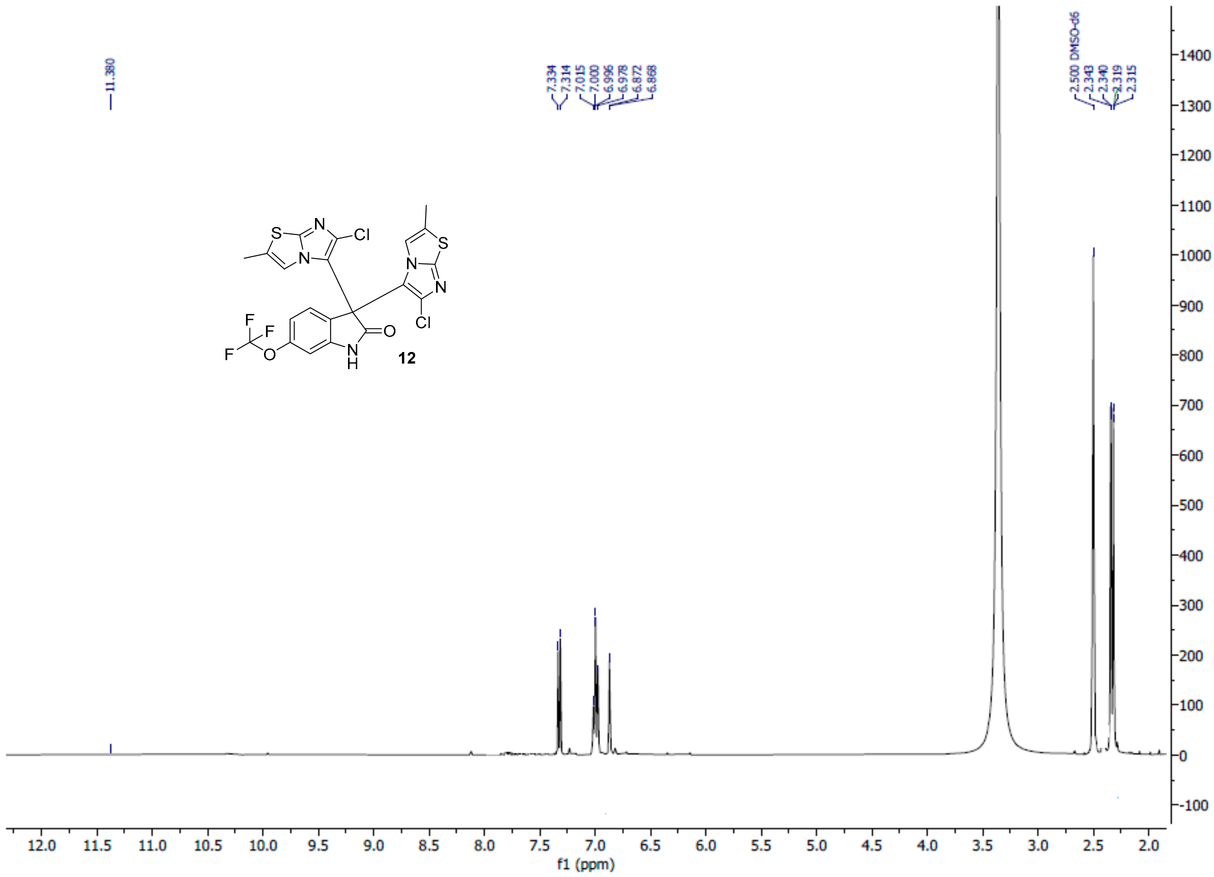
11.380
7.334
7.314
7.015
7.000
6.996
6.978
6.872
6.868
2.500 DMSO-d6
2.343
2.340
2.319
2.315
12
f1 (ppm)

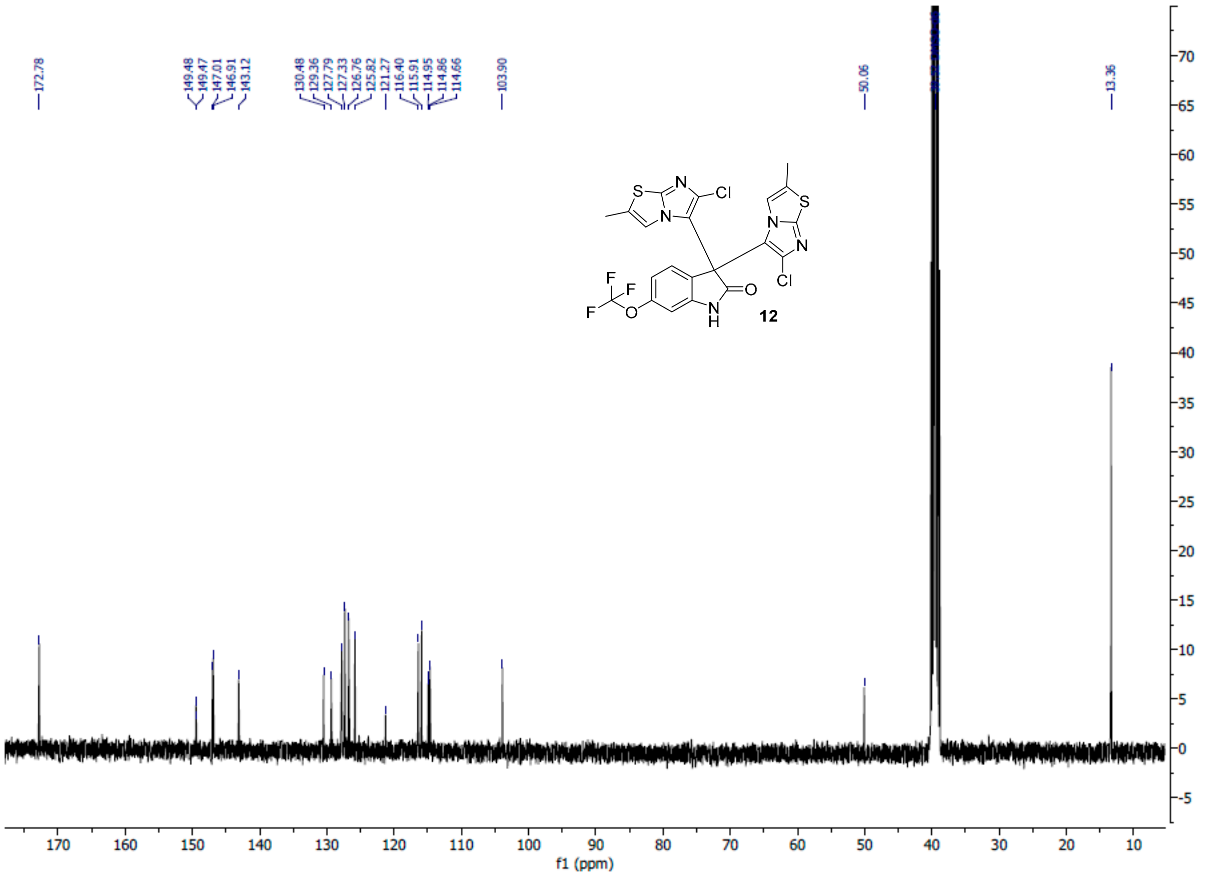
172.78
149.48
149.47
147.01
146.91
143.12
130.48
129.36
127.79
127.33
126.76
125.82
121.27
116.40
115.91
114.95
114.86
114.66
103.90
50.06
13.36
12
f1 (ppm)

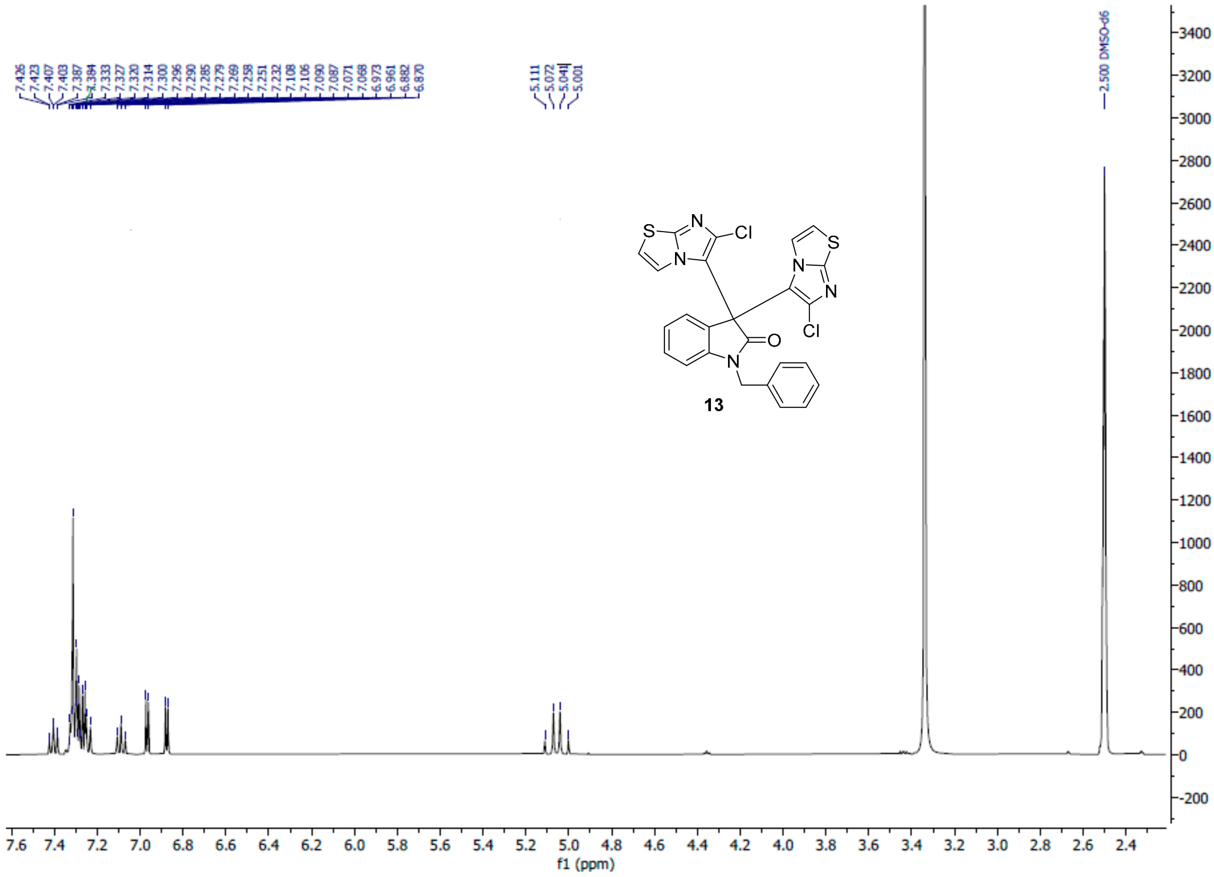
7.426
7.423
7.407
7.403
7.387
7.384
7.333
7.327
7.320
7.314
7.300
7.296
7.290
7.285
7.279
7.269
7.258
7.251
7.232
7.108
7.106
7.090
7.087
7.071
7.068
6.973
6.961
6.882
6.870
5.111
5.072
5.041
5.001
2.500 DMSO-d6
S
N
Cl
13
f1 (ppm)

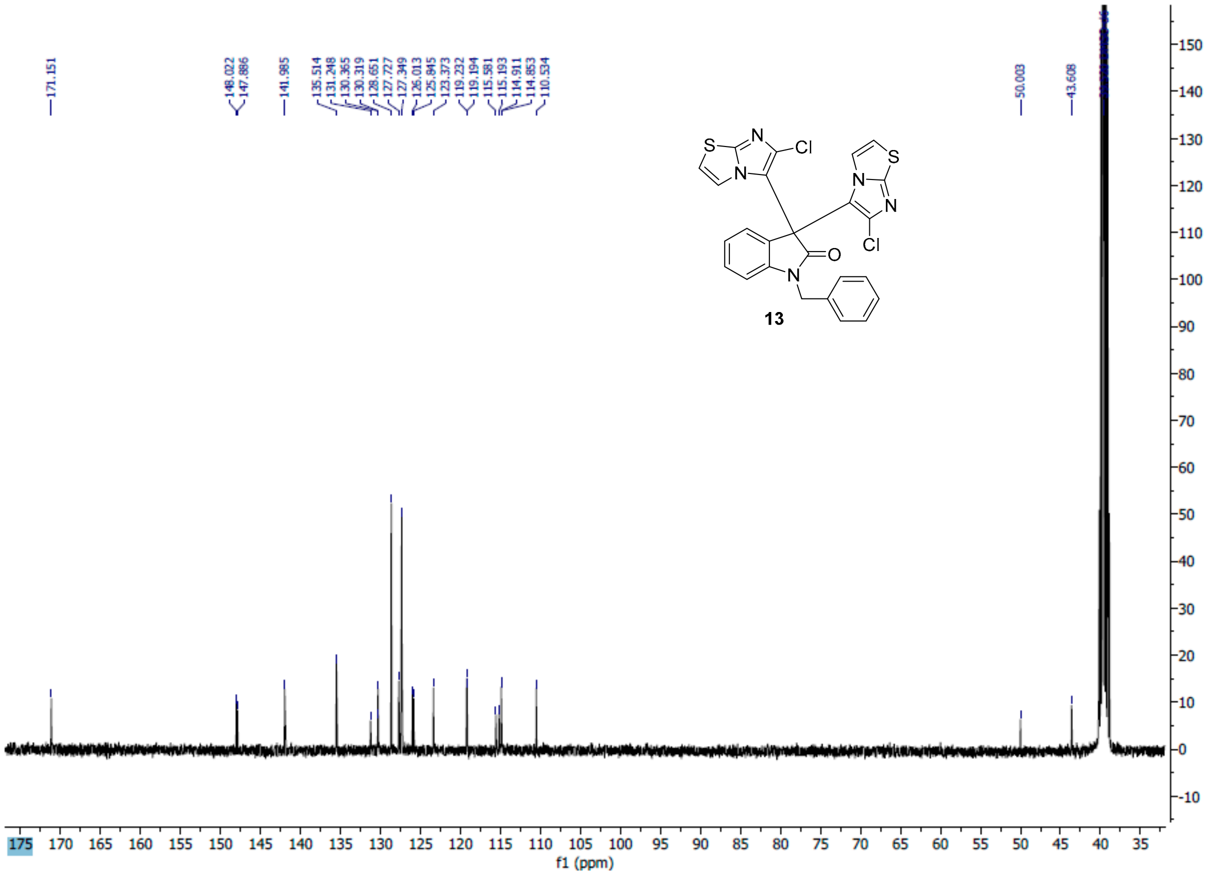
171.151
148.022
147.886
141.985
135.514
131.248
130.365
130.319
128.651
127.727
127.349
126.013
125.845
123.373
119.232
119.194
115.581
115.193
114.911
114.853
110.534
50.003
43.608
S
N
Cl
13
f1 (ppm)

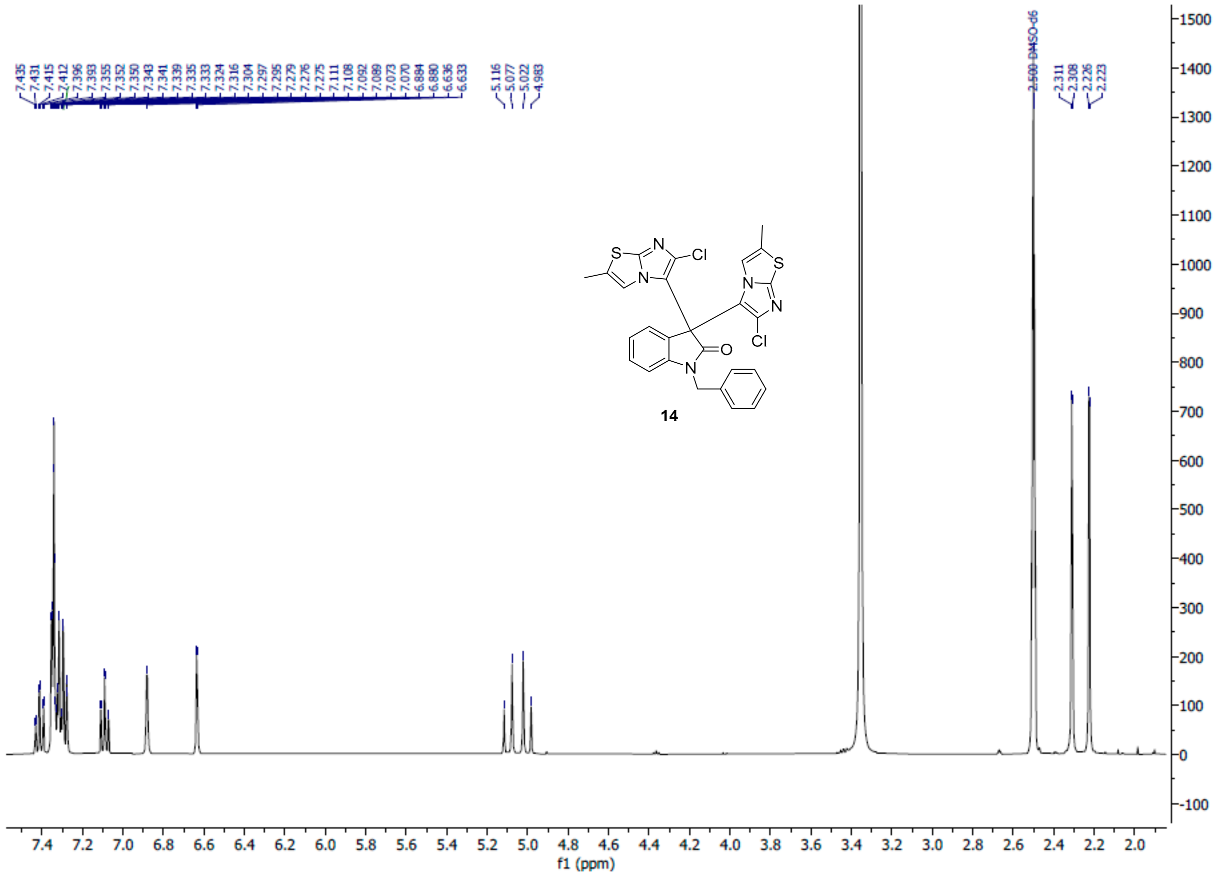
7.435 7.431 7.415 7.412 7.396 7.393 7.355 7.352 7.350 7.343 7.341 7.339 7.335 7.333 7.324 7.316 7.304 7.297 7.295 7.279 7.276 7.275 7.111 7.108 7.092 7.089 7.073 7.070 6.884 6.880 6.636 6.633
5.116 5.077 5.022 4.983
2.500 DMSO-d6
2.311 2.308 2.226 2.223
S N Cl N S N Cl O N
14
f1 (ppm)

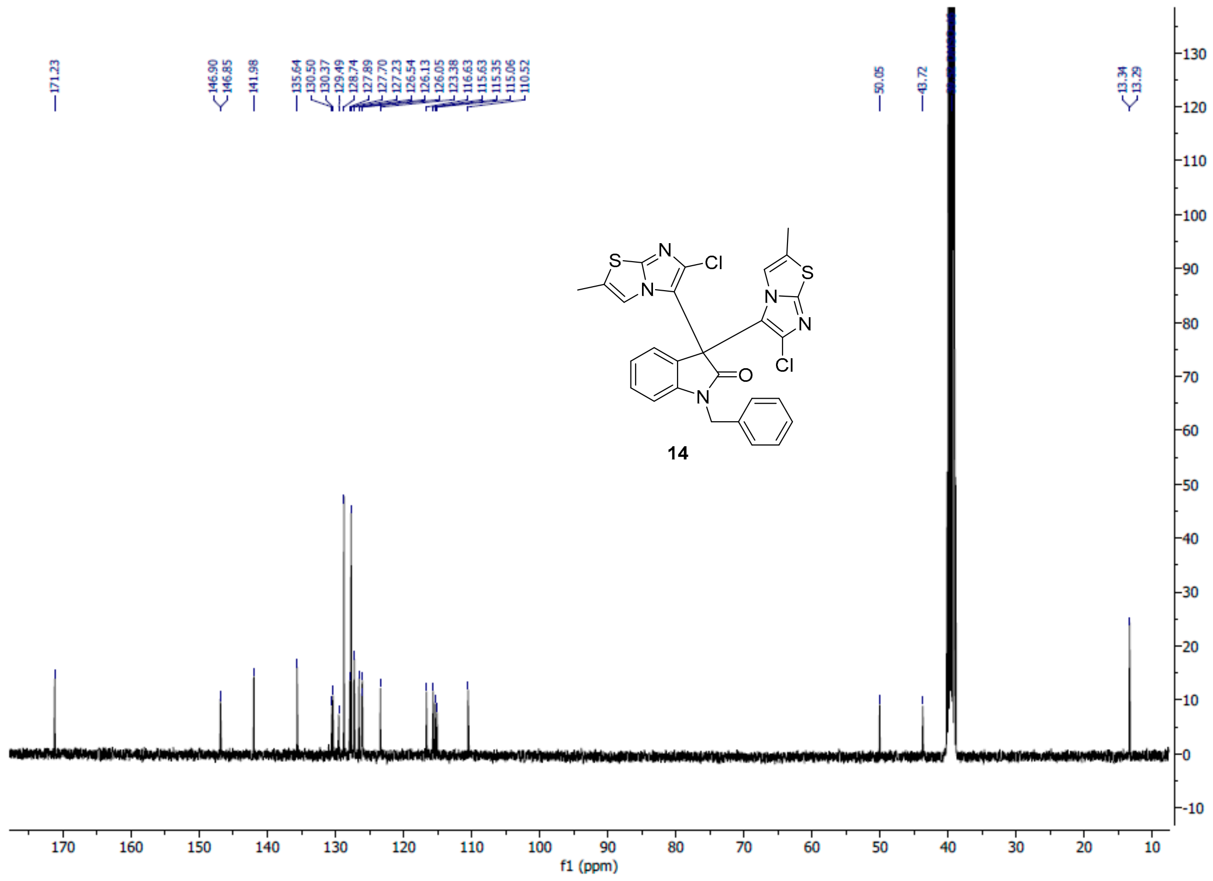
171.23
146.90 146.85 141.98
135.64 130.50 130.37 129.49 128.74 127.89 127.70 127.23 126.54 126.13 126.05 123.38 116.63 115.63 115.35 115.06 110.52
50.05 43.72
13.34 13.29
S N Cl N S N Cl O N
14
f1 (ppm)

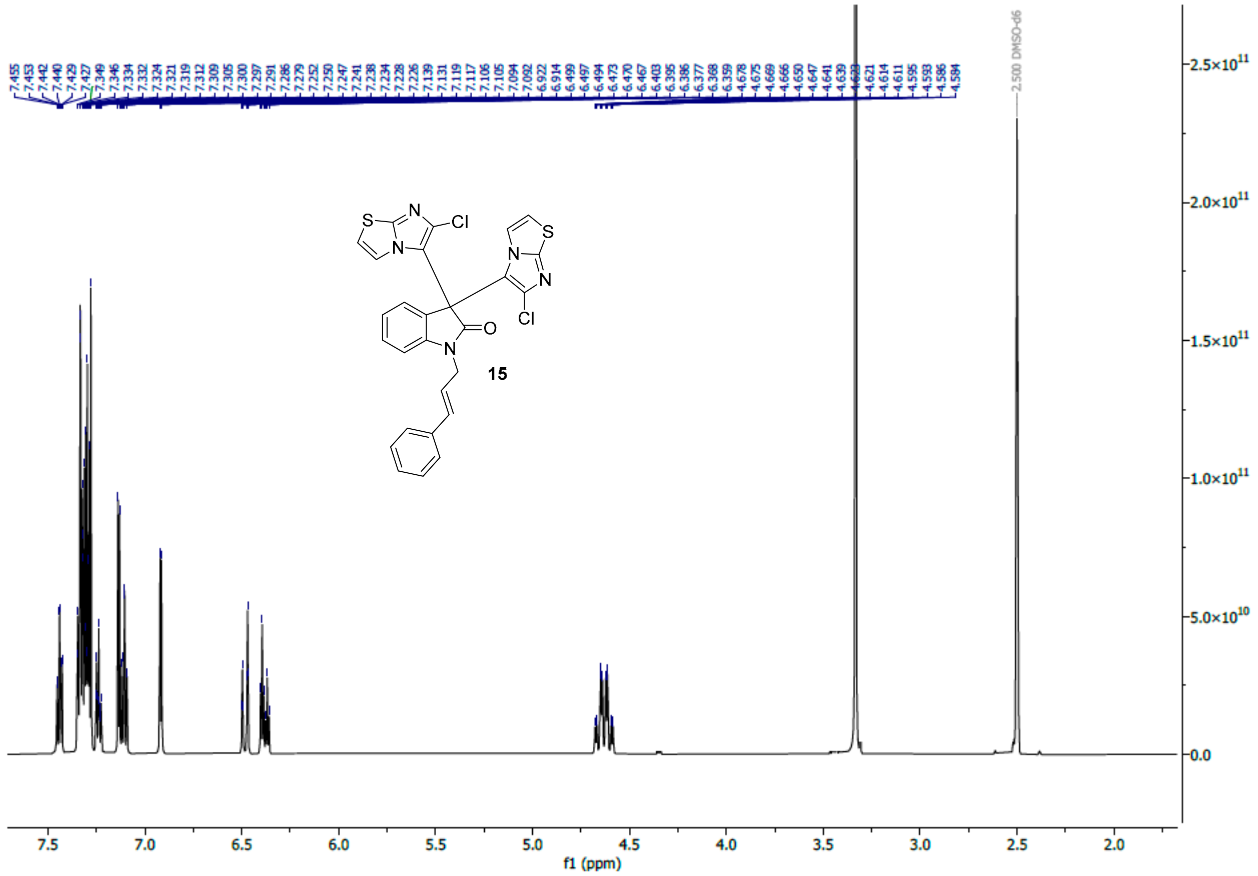
2.500 DMSO-d6
15
S
N
Cl
f1 (ppm)

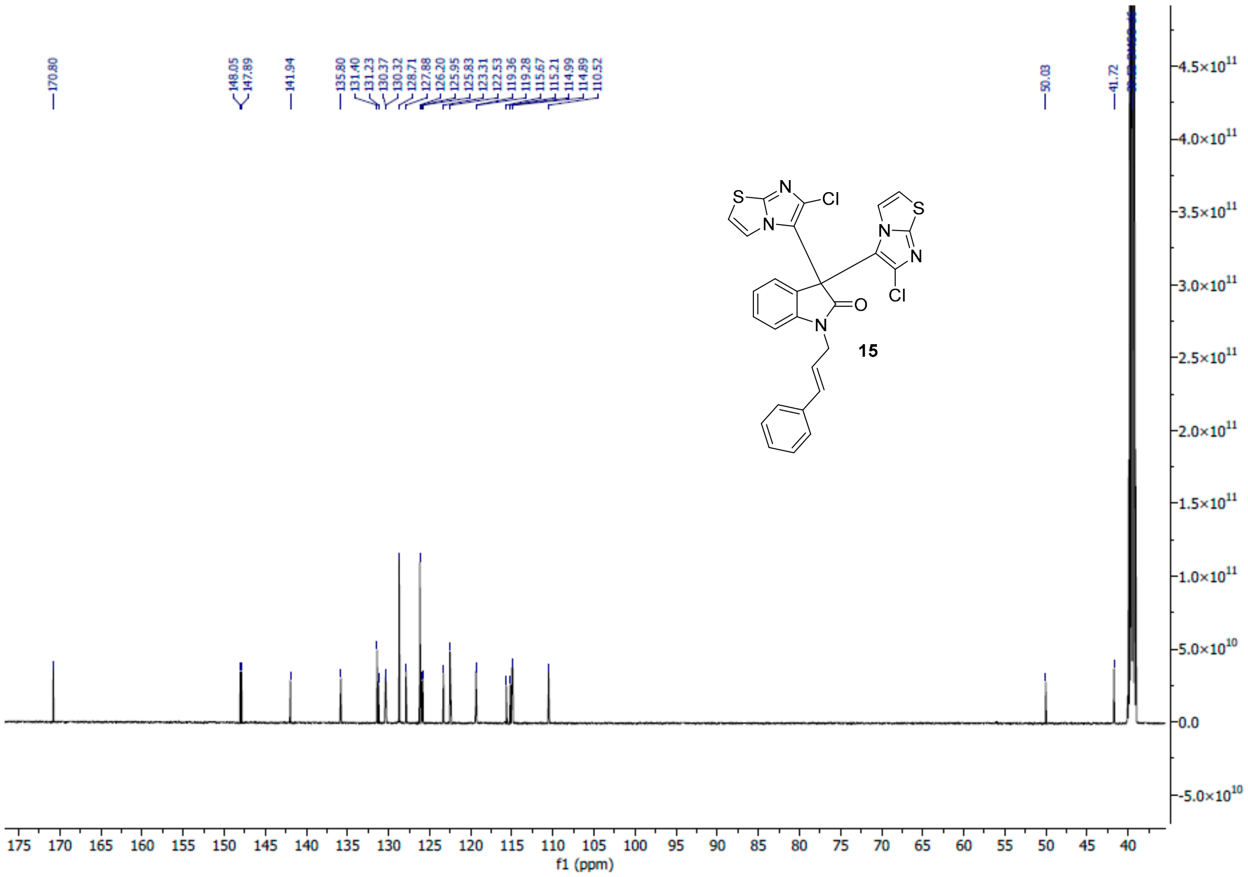
170.80
148.05
147.89
141.94
135.80
131.40
131.23
130.37
130.32
128.71
127.88
126.20
125.95
125.83
123.31
122.53
119.36
119.28
115.67
115.21
114.99
114.89
110.52
50.03
41.72
15
S
N
Cl
f1 (ppm)

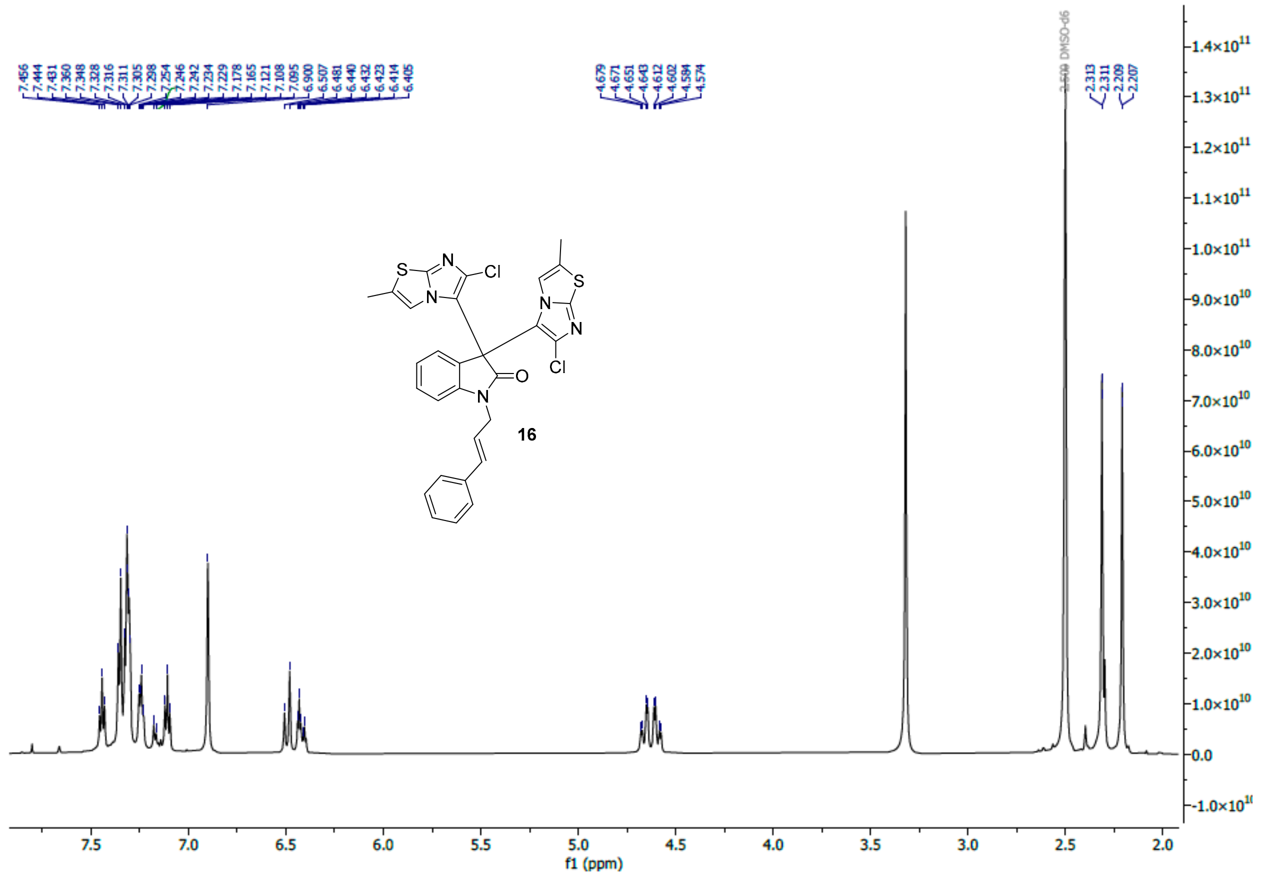

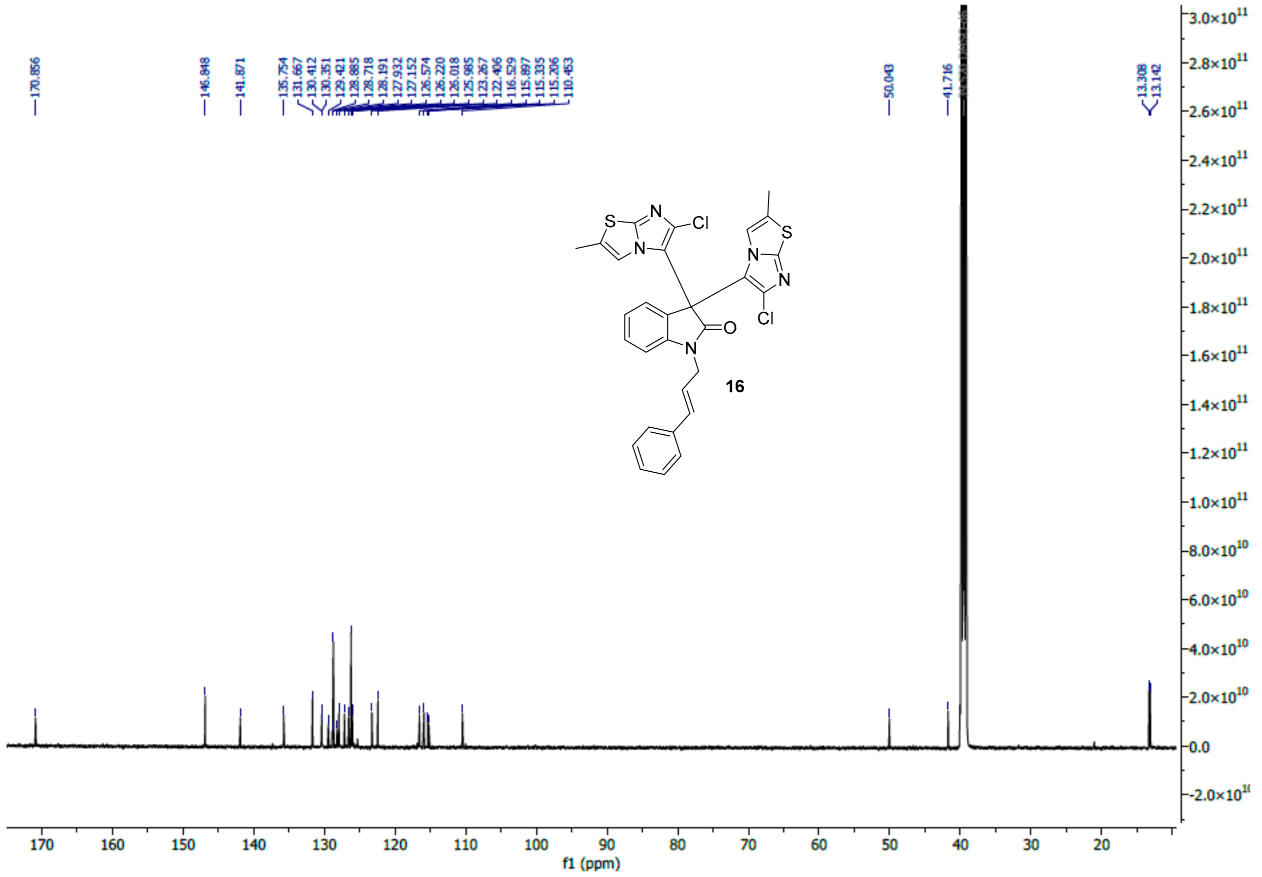

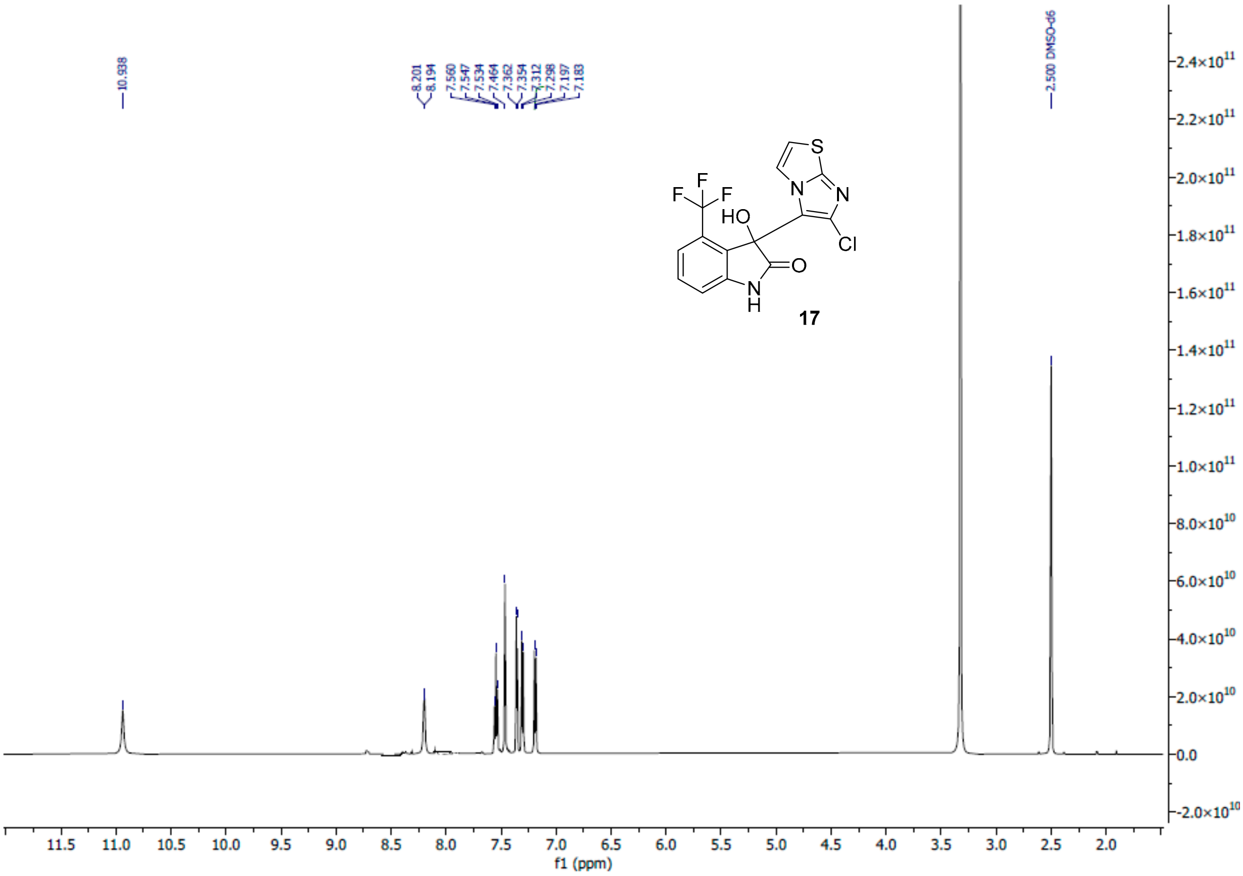
10.938
8.201
8.194
7.560
7.547
7.534
7.464
7.362
7.354
7.312
7.298
7.197
7.183
2.500 DMSO-d6
F
F
F
HO
S
N
N
Cl
O
N
H
17
f1 (ppm)

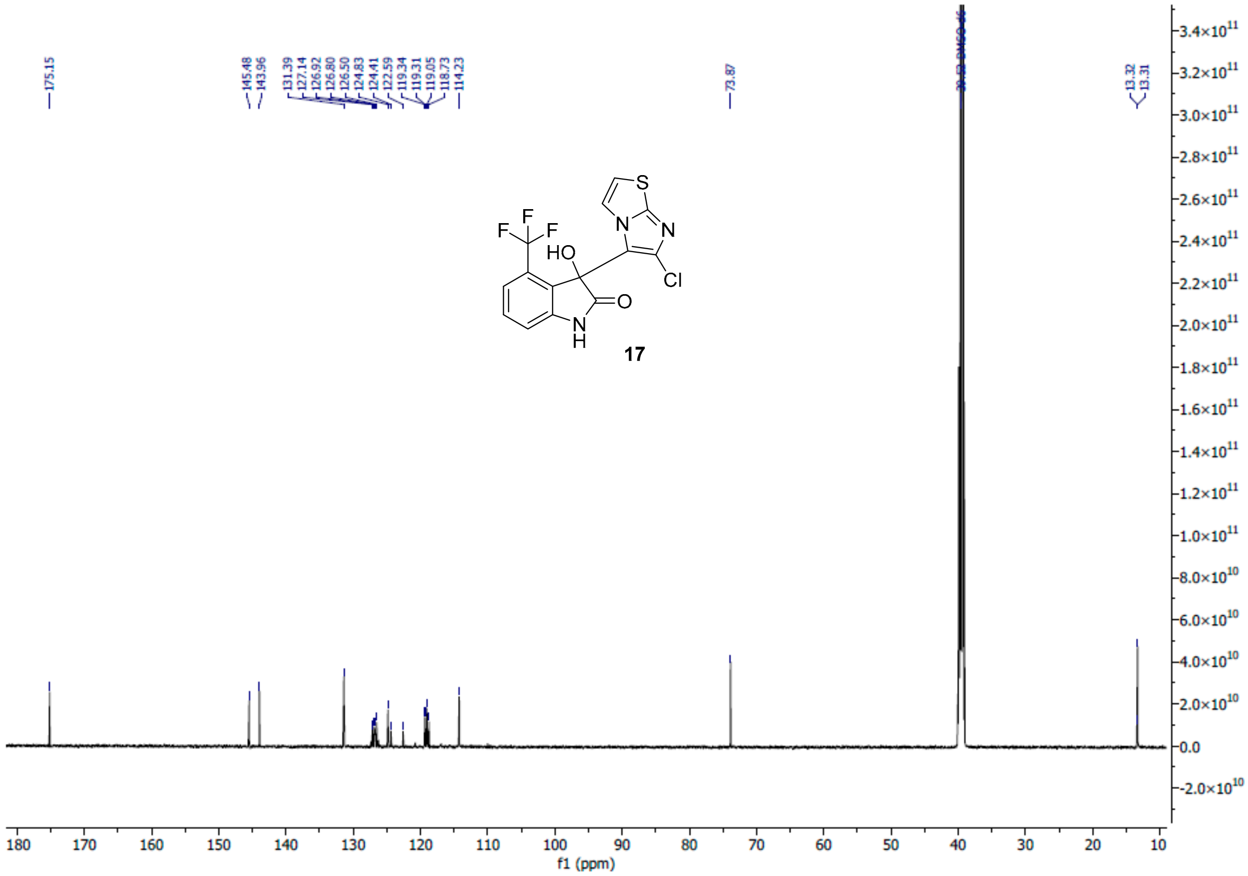
175.15
145.48
143.96
131.39
127.14
126.92
126.80
126.50
124.83
124.41
122.59
119.34
119.31
119.05
118.73
114.23
73.87
13.32
13.31
F
F
F
HO
S
N
N
Cl
O
N
H
17
f1 (ppm)

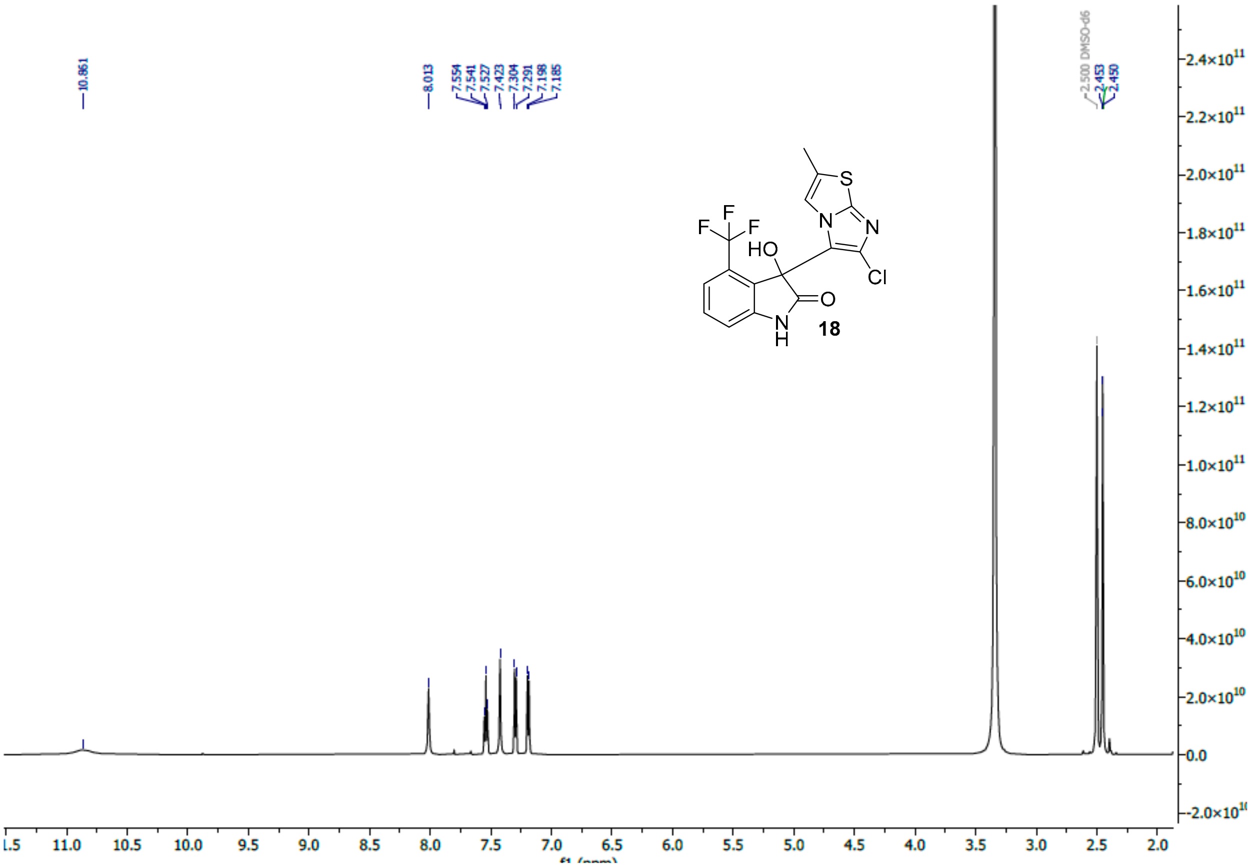
10.861
8.013
7.554
7.541
7.527
7.423
7.304
7.291
7.198
7.185
2.500 DMSO-d6
2.453
2.450
HO
N
S
Cl
O
H
18
f1 (ppm)

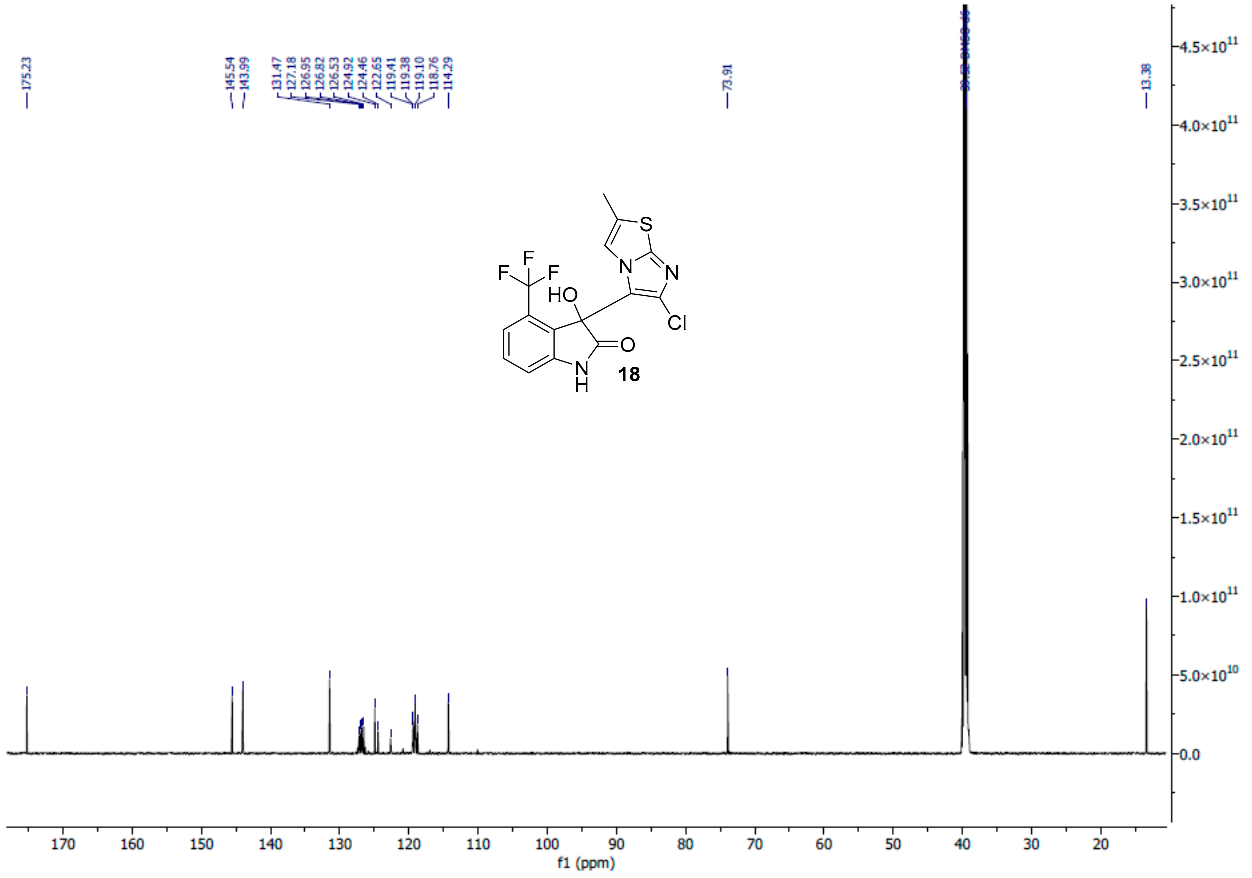
175.23
145.54
143.99
131.47
127.18
126.95
126.82
126.53
124.92
124.46
122.65
119.41
119.38
119.10
118.76
114.29
73.91
13.38
HO
N
S
Cl
O
H
18
f1 (ppm)

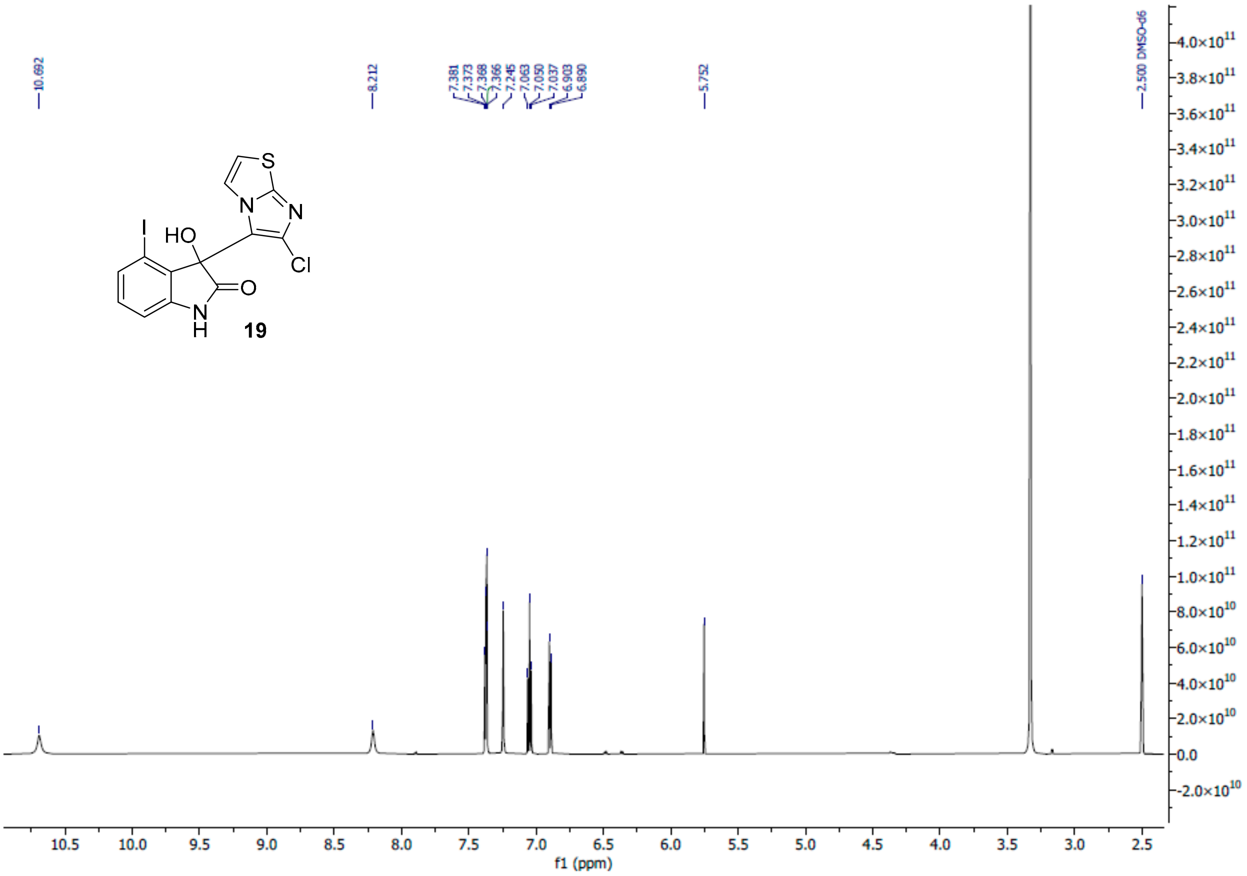
10.692
8.212
7.381
7.373
7.368
7.366
7.245
7.063
7.050
7.037
6.903
6.890
5.752
2.500 DMSO-d6
HO
I
S
N
N
Cl
O
N
H
19
f1 (ppm)

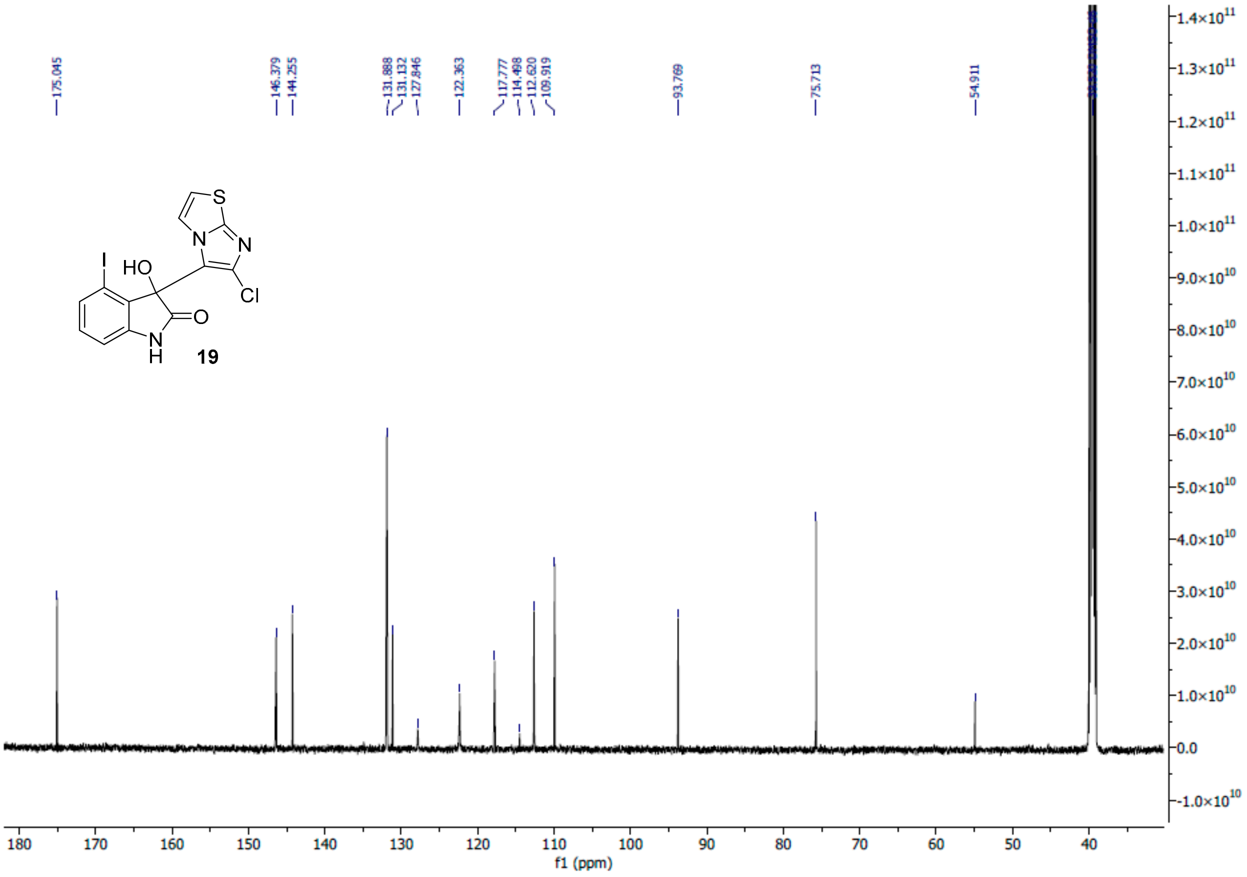
175.045
146.379
144.255
131.888
131.132
127.846
122.363
117.777
114.498
112.620
109.919
93.769
75.713
54.911
HO
I
S
N
N
Cl
O
N
H
19
f1 (ppm)

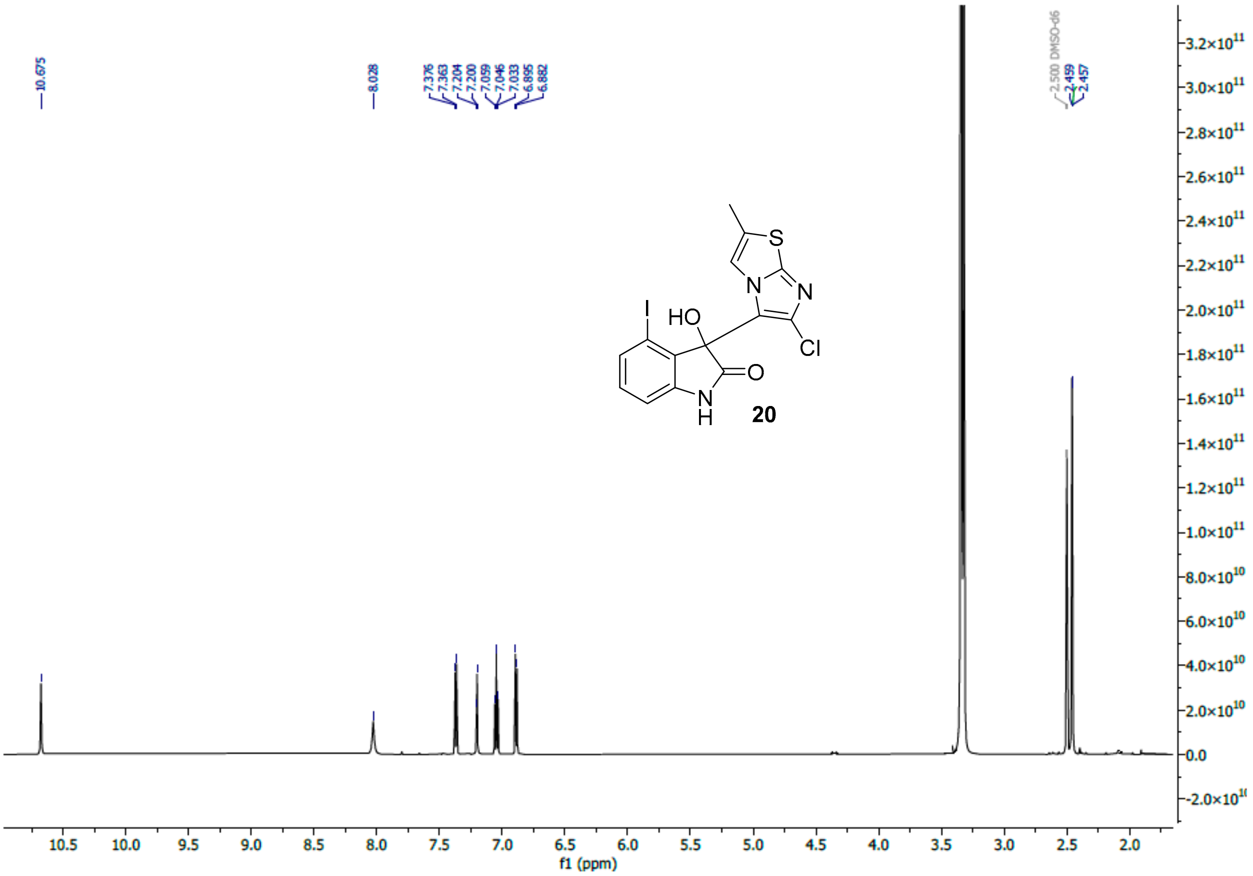
10.675
8.028
7.376
7.363
7.204
7.200
7.059
7.046
7.033
6.895
6.882
2.500 DMSO-d6
2.459
2.457
HO
S
N
Cl
O
H
20
f1 (ppm)

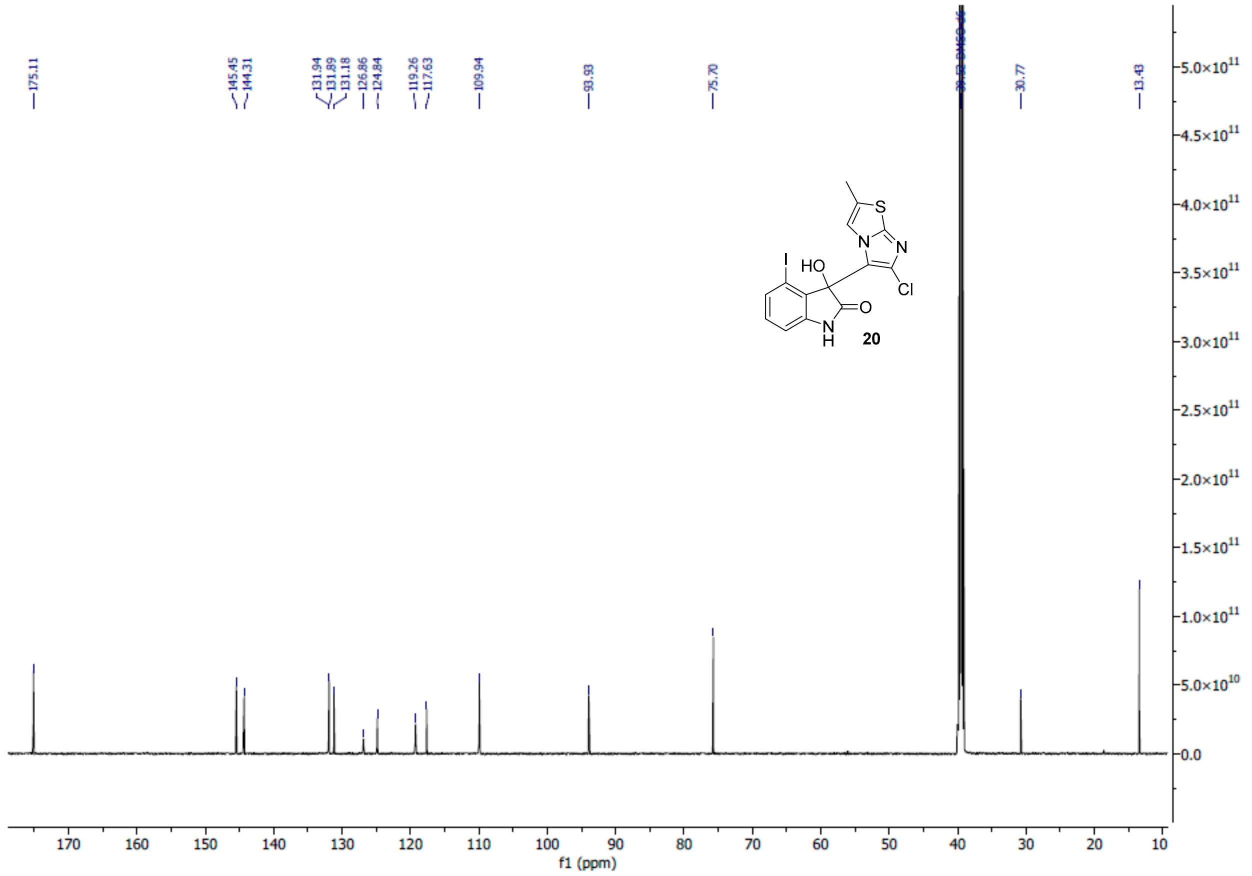
175.11
145.45
144.31
131.94
131.89
131.18
126.86
124.84
119.26
117.63
109.94
93.93
75.70
30.77
13.43
HO
S
N
Cl
O
H
20
f1 (ppm)

Figure S2. A) Multiple sequence alignment (obtained with Chimera, using the Match-Align tool) of the primary sequences of PDBs 6LL6 (from *E. coli*), 3VOB (from *S. aureus*), 8GZV (from *K. pneumoniae)*, 2VAW (from *P. aeruginosa*), AF-B0VNZ4-F1 (predicted for *A. baumannii*) and AF-A0A0A5PLG7-F1 (predicted for *E. hormaechei).* B) Superimposition of 3D structures of PDBs 6LL6, 3VOB, 8GZV, 2VAW, AF-B0VNZ4-F1 and AF-A0A0A5PLG7-F1 in blue, red, yellow, green, magenta and cyan, respectively.

A

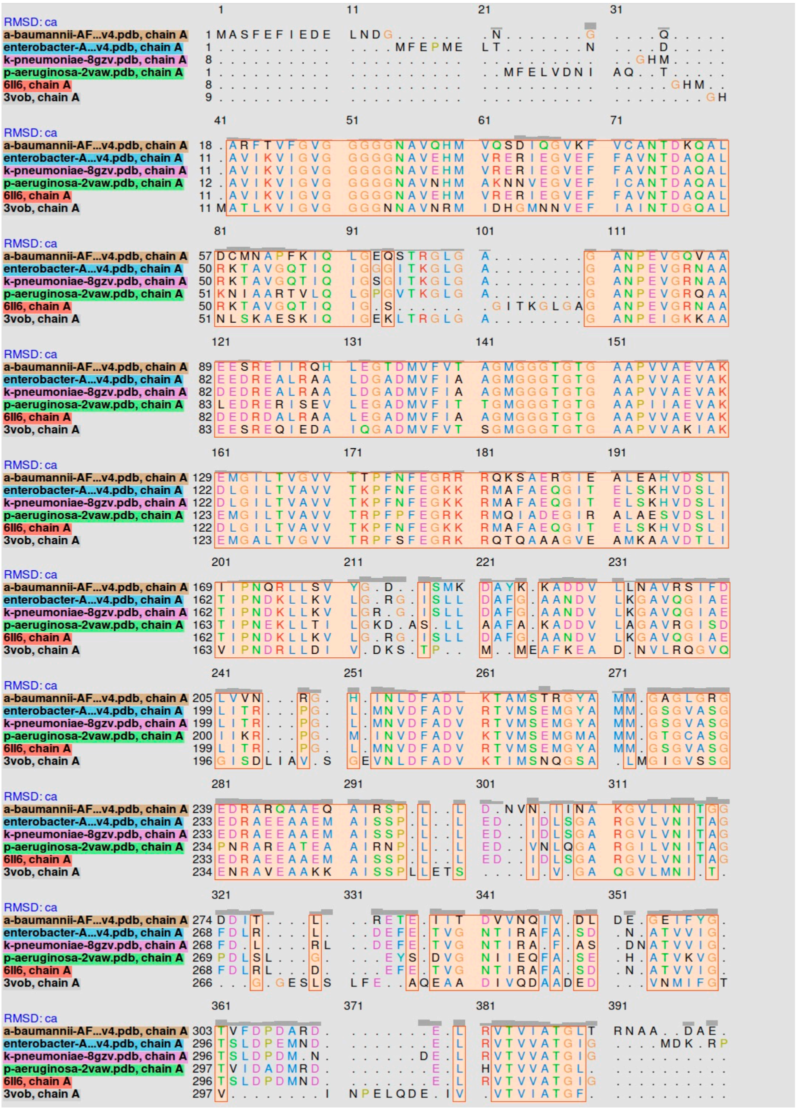

B

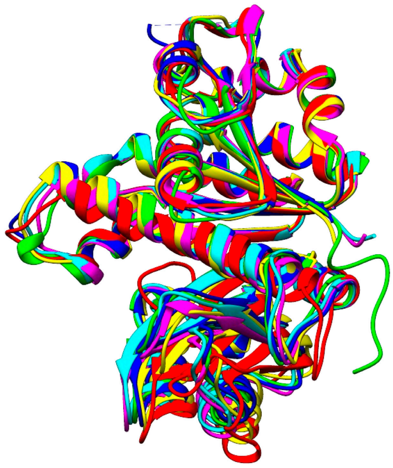

Table S1. Hemolytic activity of derivative **11** on hRBCs (range concentration: 100 -1.56 µM)**.** Data are the mean percentage values and standard deviations of the hemoglobin content measured at 405 nm in the supernatants of the treated cells.

Compound 11	Percentage values[§]
**100 µM**	1.1 ± 0.4
**50 µM**	0.7 ± 1.5
**25 µM**	1.8 ± 1.5
**12.5 µM**	2.2 ± 0.5
**6.25 µM**	2.3 ± 0.2
**3.125 µM**	1.1 ± 1.2
**1.56 µM**	0.9 ± 1.8

[§] data are relative to untreated hRBCs (incubated with PBS) and hRBCs incubated with 1% Triton X-100.

Table S2. Antibiotic-resistance profile of the clinical isolates of S. *aureus*

Clinical isolate	Antibiotic-resistance profile
**MRSA 1**[§]	GEN^{S}, LVX^{R}, **OX**^{R}, **P**^{R}, TE^{S}, TEC^{S}, SXT^{S}, VA^{S}
**MRSA 2**[§]	GEN^{S}, LVX^{R}, **OX**^{R}, **P**^{R}, TE^{S}, TEC^{S}, SXT^{S}, VA^{S}
**MRSA 3**[§]	**CM**^{R}**, E**^{R}**,** GEN^{S}, **LVX**^{R}, **OX**^{R}, **P**^{R}, TEC^{S}, TE^{S}, SXT^{S}, VA^{S}
**MRSA 4**[§]	CM^{S}, E^{S}, GEN^{S}, **LVX**^{R}, **OX**^{R}, **P**^{R}, TE^{S}, TEC^{S}, SXT^{S}, VA^{S}
**MRSA 5**[§]	CM^{S}, E^{S}, GEN^{S}, **LVX**^{R}, **OX**^{R}, **P**^{R}, TE^{S}, TEC^{S}, SXT^{S}, VA^{S}

CM = Clindamicyn; E = Erythromycin; GEN = Gentamicin; P = Penicillin; LVX = Levofloxacin; OX = Oxacillin; TE = Tetracycline; TEC = Teicoplanin; SXT = Trimethoprim/Sulfamethoxazole; VA = Vancomycin
R = Resistant; S = Susceptible; I = Intermediate, as defined following the EUCAST guidelines
[§]*Staphylococcus* species resistant to oxacillin were declared, by convention, methicillin-resistant.

Figure S3. A) Root of mean squared deviation (RMSD), B) Root of mean squared fluctuation RMSF, C) Gyration Radius (RG) and D) Solvent Accessible Surface Area (SASA) and ΔSASA ([$SASA_{protein}$+$SASA_{compound11}$]-$SASA_{complex}$) obtained by CPPTRAJ post-processing analysis of 100ns MD simulation of compound **11** in the interdomain cleft. All the analysis are carried out on 5000 snapshots obtained from the simulation.

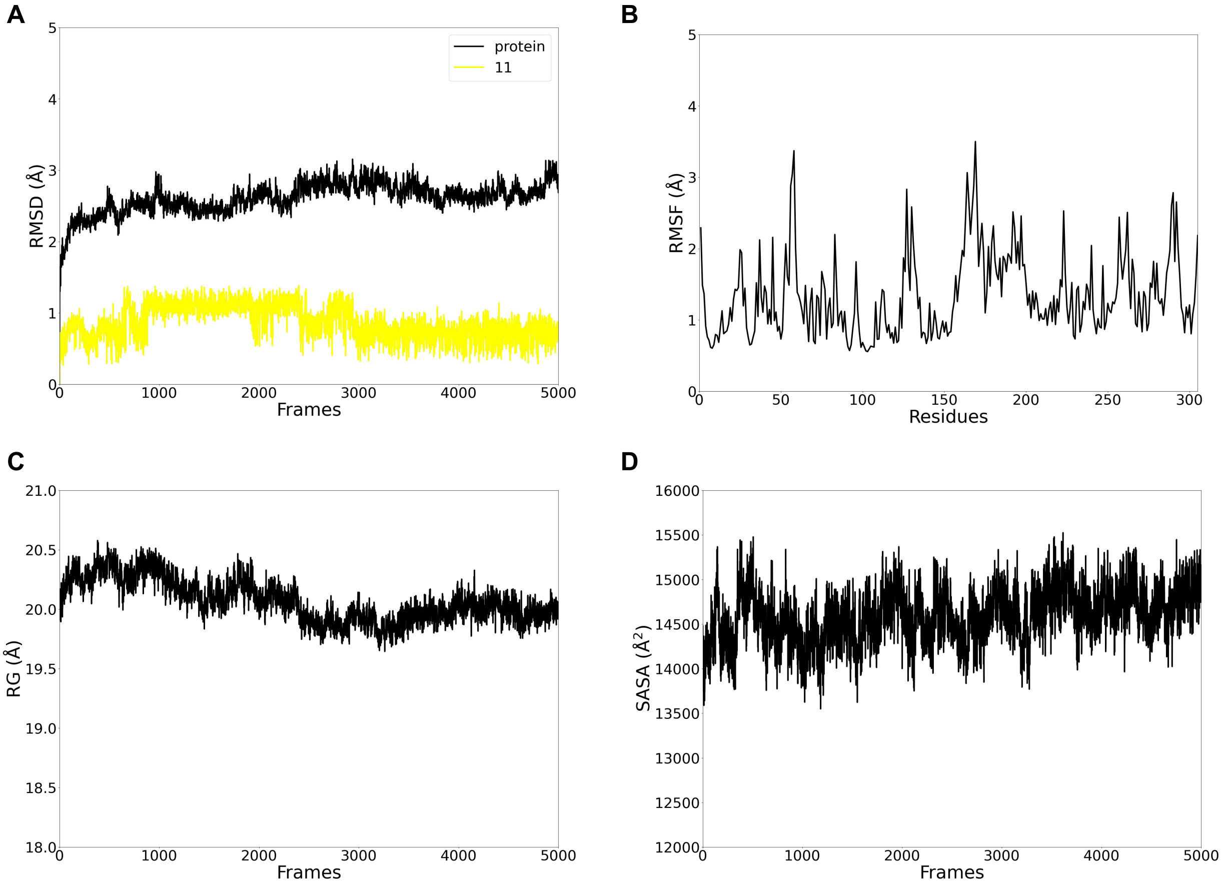

Table S3. Inter-molecular hydrogen bond analysis carried out on the MD simulation of compound **11** in the interdomain cleft.

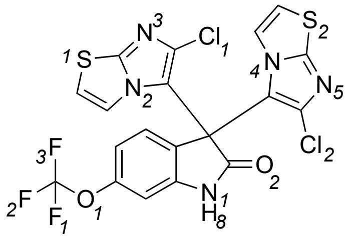

Acceptor	DonorH	Donor	%	Avg Distance	Avg Angle
GLN_181@O	C11_305@H8	C11_305@N1	36,2	2,8	156,1
GLY_185@O	C11_305@H8	C11_305@N1	30,2	2,8	150,8
GLN_181@OE1	C11_305@H8	C11_305@N1	13,1	2,8	156,1
C11_305@N3	SER_193@HG	SER_193@OG	1,3	2,9	161,9
C11_305@O2	GLN_184@HE22	GLN_184@NE2	1,0	2,9	160,7
C11_305@N5	THR_298@HG1	THR_298@OG1	0,5	2,9	154,9
ASP_188@OD2	C11_305@H8	C11_305@N1	0,3	2,8	163,7
C11_305@O2	GLN_184@HE21	GLN_184@NE2	0,2	2,9	155,4
C11_305@N3	ASN_252@HD22	ASN_252@ND2	0,2	2,9	148,9
C11_305@O2	GLN_181@HE21	GLN_181@NE2	0,1	2,9	161,1
C11_305@O2	GLN_181@HE22	GLN_181@NE2	0,1	2,8	155,7
ASP_188@OD1	C11_305@H8	C11_305@N1	0,1	2,8	154,3

Figure S4. A) Root of mean squared deviation (RMSD), B) Root of mean squared fluctuation RMSF, C) Gyration Radius (RG) and D) Solvent Accessible Surface Area (SASA) obtained by CPPTRAJ post-processing analysis of 100ns MD simulation of compound **11** in the GPT-binding pocket. All the analysis are carried out on 5000 snapshots obtained from the simulation.

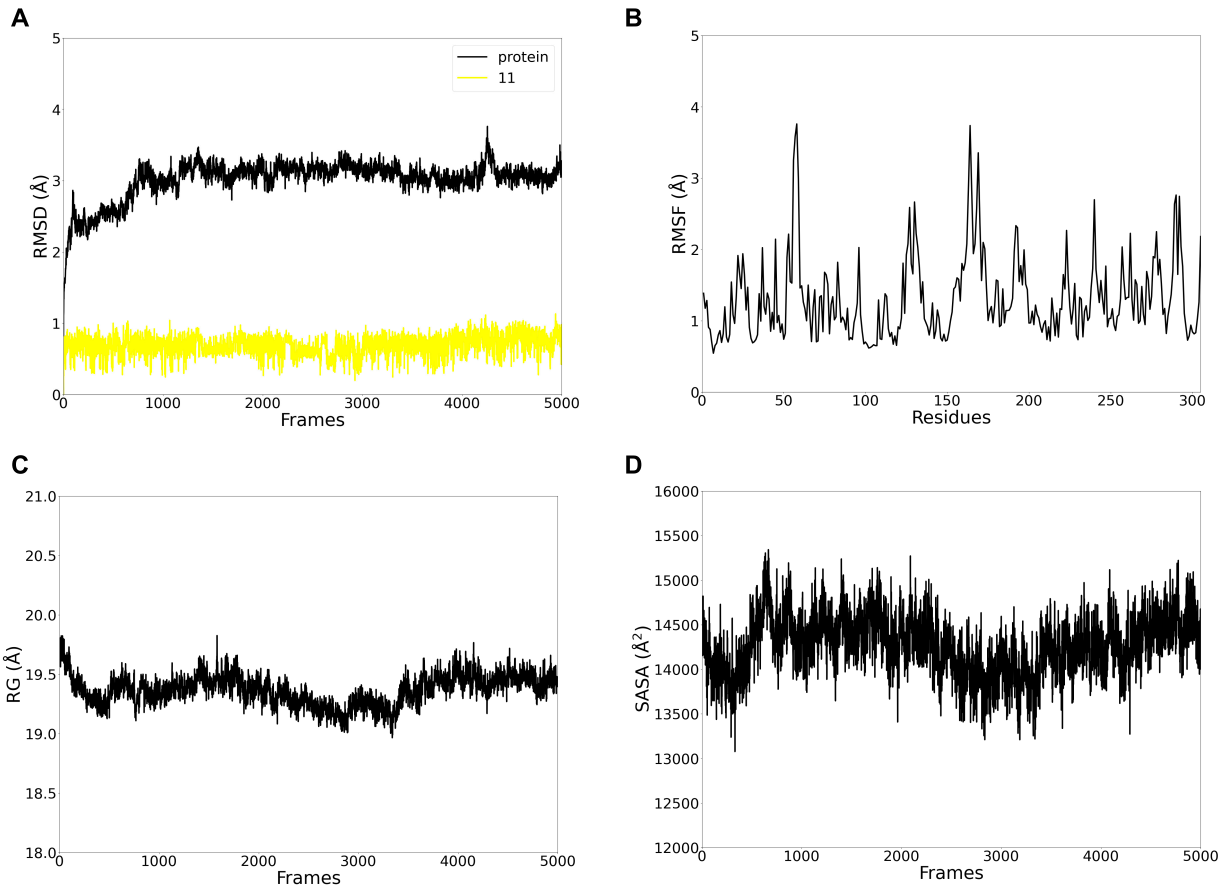

Table S4. Inter-molecular hydrogen bond analysis carried out on the MD simulation of compound **11** in the GTP-binding pocket.

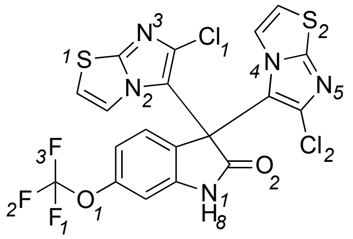

Acceptor	DonorH	Donor	%	Avg Distance	Avg Angle
C11_305@N5	ASN_14@HD22	ASN_14@ND2	14,2	2,9	159,1
C11_305@N5	GLY_11@H	GLY_11@N	6,2	2,9	159,9
ASP_176@OD1	C11_305@H8	C11_305@N1	5,2	2,8	164,3
ASP_176@OD2	C11_305@H8	C11_305@N1	2,4	2,8	164,0
C11_305@N3	ASN_155@HD21	ASN_155@ND2	2,2	2,9	152,3
ASN_14@OD1	C11_305@H8	C11_305@N1	0,7	2,8	147,7
C11_305@O2	ARG_18@HH12	ARG_18@NH1	0,3	2,8	147,8
C11_305@N3	THR_122@HG1	THR_122@OG1	0,3	2,9	147,3
PHE_172@O	C11_305@H8	C11_305@N1	0,1	2,9	141,4

Figure S5. A) Root of mean squared deviation (RMSD), B) Root of mean squared fluctuation RMSF, C) Gyration Radius (RG) and D) Solvent Accessible Surface Area (SASA) obtained by CPPTRAJ post-processing analysis of 100ns MD simulation of apo-FtsZ. All the analysis are carried out on 5000 snapshots obtained from the simulation.

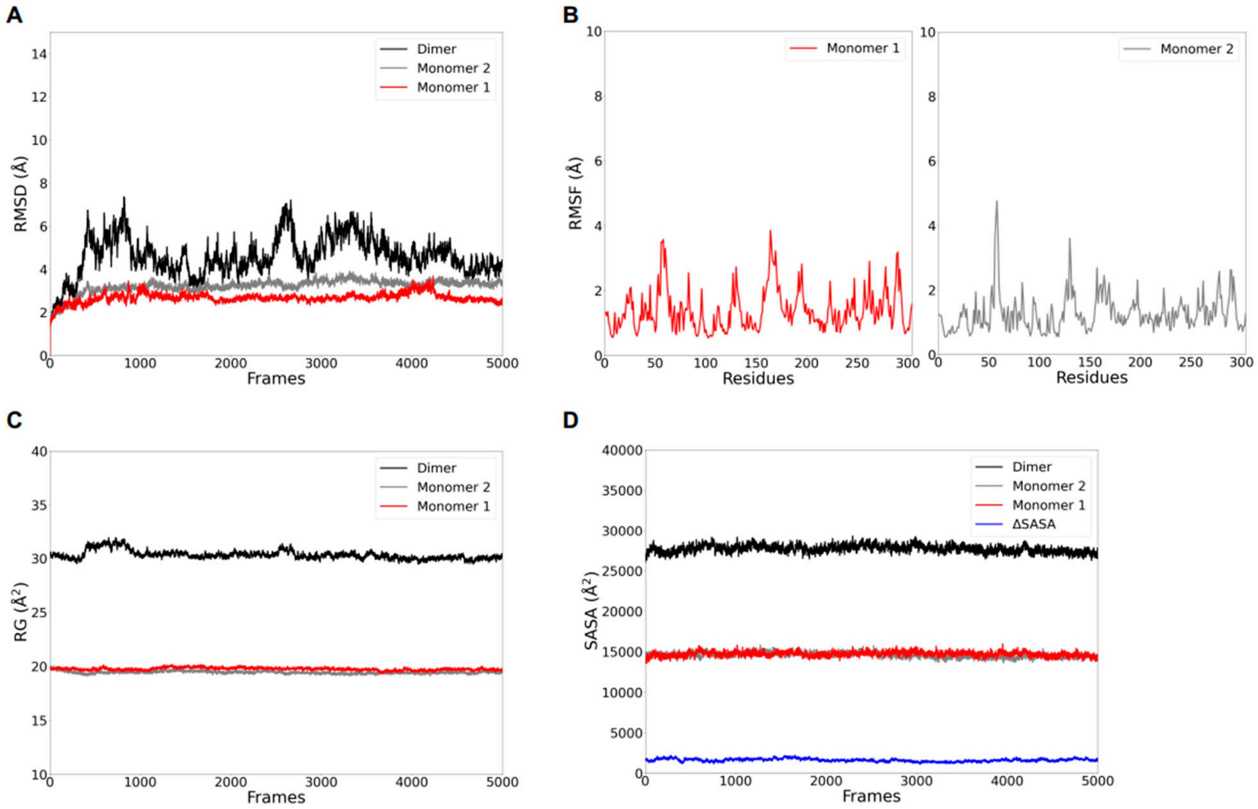

Table S5. Intra-molecular hydrogen bond analysis carried out on monomer 1 of the MD simulation of apo-FtsZ dimer. Only occurrences higher than 50% are reported.

Acceptor	DonorH	Donor	%
ALA_201@O	THR_205@HG1	THR_205@OG1	89,6
ILE_300@O	LEU_250@H	LEU_250@N	85,5
PHE_89@O	ILE_6@H	ILE_6@N	85,0
PHE_200@O	LYS_204@H	LYS_204@N	83,5
THR_117@O	ASP_148@H	ASP_148@N	82,8
VAL_120@O	THR_91@HG1	THR_91@OG1	82,1
ILE_253@O	THR_285@HG1	THR_285@OG1	81,7
GLY_119@O	ILE_151@H	ILE_151@N	81,4
VAL_8@O	SER_92@HG	SER_92@OG	79,0
VAL_118@O	VAL_90@H	VAL_90@N	76,3
LEU_116@O	VAL_88@H	VAL_88@N	73,9
ARG_130@O	THR_134@HG1	THR_134@OG1	73,7
ILE_66@O	ALA_70@H	ALA_70@N	72,8

VAL_88@O	VAL_118@H	VAL_118@N	72,8
ASP_163@O	THR_166@HG1	THR_166@OG1	72,6
GLY_218@O	VAL_297@H	VAL_297@N	72,4
ILE_32@O	GLY_9@H	GLY_9@N	71,6
ALA_115@O	THR_117@HG1	THR_117@OG1	70,8
LEU_250@O	ILE_300@H	ILE_300@N	70,4
ILE_30@O	GLY_7@H	GLY_7@N	70,2
ALA_70@O	SER_74@HG	SER_74@OG	69,6
GLU_28@O	VAL_5@H	VAL_5@N	69,6
ASN_17@OD1	LYS_43@H	LYS_43@N	69,5
VAL_286@O	THR_254@HG1	THR_254@OG1	69,0
ASN_177@O	GLN_181@H	GLN_181@N	68,3
GLY_211@O	LEU_150@H	LEU_150@N	68,0
VAL_121@O	ILE_153@H	ILE_153@N	67,8
ASP_176@OD1	ARG_18@HE	ARG_18@NE	67,4
ASP_293@O	ARG_225@HH11	ARG_225@NH1	67,4
GLY_9@O	ASN_13@H	ASN_13@N	67,2
THR_34@OG1	THR_98@HG1	THR_98@OG1	66,6
VAL_297@O	GLY_218@H	GLY_218@N	65,6
THR_149@O	GLY_119@H	GLY_119@N	64,9
ALA_136@O	VAL_140@H	VAL_140@N	64,5
ARG_130@O	THR_134@H	THR_134@N	64,4
GLY_139@O	MET_143@H	MET_143@N	64,0
VAL_140@O	LYS_144@H	LYS_144@N	63,2
VAL_296@O	THR_254@H	THR_254@N	62,7
ILE_6@O	THR_91@H	THR_91@N	62,6
GLY_248@O	THR_302@H	THR_302@N	61,6
THR_91@O	VAL_8@H	VAL_8@N	61,3
PRO_64@O	LYS_68@H	LYS_68@N	61,0
VAL_249@O	ILE_282@H	ILE_282@N	60,8
GLU_277@O	VAL_279@H	VAL_279@N	60,7
ASN_252@O	THR_298@H	THR_298@N	60,6
ALA_107@O	LYS_111@H	LYS_111@N	60,4
LYS_4@O	PHE_89@H	PHE_89@N	59,9
ARG_180@O	GLN_184@H	GLN_184@N	59,6
GLY_99@O	ALA_103@H	ALA_103@N	59,4
GLY_255@O	ASN_288@H	ASN_288@N	59,0
ASP_176@O	ARG_180@H	ARG_180@N	59,0
LYS_108@O	GLU_112@H	GLU_112@N	59,0
ILE_32@O	ASN_13@HD21	ASN_13@ND2	58,8
THR_302@O	GLN_247@H	GLN_247@N	58,1
GLN_181@O	GLY_185@H	GLY_185@N	57,8
ALA_301@O	LEU_214@H	LEU_214@N	57,5
VAL_90@O	VAL_120@H	VAL_120@N	56,6
THR_254@O	VAL_296@H	VAL_296@N	56,5
LYS_131@O	GLN_135@H	GLN_135@N	56,2
SER_220@O	ILE_295@H	ILE_295@N	56,1
GLY_216@O	VAL_299@H	VAL_299@N	55,5
GLY_51@O	THR_55@H	THR_55@N	55,4

VAL_5@O	ILE_30@H	ILE_30@N	55,0
VAL_203@O	MET_207@H	MET_207@N	54,8
MET_215@O	SER_235@HG	SER_235@OG	54,7
LYS_204@O	SER_208@HG	SER_208@OG	54,4
ILE_153@O	ARG_123@H	ARG_123@N	54,2
ILE_282@O	MET_251@H	MET_251@N	52,3
VAL_105@O	ILE_109@H	ILE_109@N	52,1
ASN_224@O	GLU_228@H	GLU_228@N	51,1
ASP_86@O	LEU_116@H	LEU_116@N	50,6
ARG_225@O	ALA_229@H	ALA_229@N	50,4
ARG_75@O	GLU_79@H	GLU_79@N	50,2
GLU_263@OE2	ARG_225@HH21	ARG_225@NH2	50,1

Table S6. Intra-molecular hydrogen bond analysis carried out on monomer 2 of the MD simulation of apo-FtsZ dimer. Only occurrences higher than 50% are reported. To the residue number in monomer 2, 304 units needs to be subtracted to obtain the original residue number (ASP_467 = ASP_163).

Acceptor	DonorH	Doron	%
ASP_467@O	THR_470@HG1	THR_470@OG1	94,5
VAL_424@O	THR_395@HG1	THR_395@OG1	88,1
THR_421@O	ASP_452@H	ASP_452@N	85,3
LEU_554@O	ILE_604@H	ILE_604@N	85,1
LYS_477@O	ARG_484@HH12	ARG_484@NH1	84,2
ILE_604@O	LEU_554@H	LEU_554@N	83,8
PHE_393@O	ILE_310@H	ILE_310@N	83,6
ILE_370@O	ALA_374@H	ALA_374@N	83,5
GLY_423@O	ILE_455@H	ILE_455@N	82,9
GLY_559@O	ASN_592@H	ASN_592@N	81,6
GLY_313@O	ASN_317@H	ASN_317@N	81,3
ALA_505@O	THR_509@HG1	THR_509@OG1	80,5
THR_558@O	VAL_600@H	VAL_600@N	79,4
PHE_504@O	LYS_508@H	LYS_508@N	78,6
GLY_522@O	VAL_601@H	VAL_601@N	78,6
LEU_420@O	VAL_392@H	VAL_392@N	78,4
GLY_588@O	THR_589@HG1	THR_589@OG1	75,6
ILE_336@O	GLY_313@H	GLY_313@N	75,6
VAL_590@O	THR_558@HG1	THR_558@OG1	75,5
VAL_425@O	ILE_457@H	ILE_457@N	74,2
GLU_332@O	VAL_309@H	VAL_309@N	74,0
VAL_603@O	GLY_520@H	GLY_520@N	73,9
GLY_520@O	VAL_603@H	VAL_603@N	73,5
GLN_488@O	ASP_492@H	ASP_492@N	73,1
ALA_374@O	SER_378@HG	SER_378@OG	72,8
ALA_419@O	THR_421@HG1	THR_421@OG1	71,6
VAL_312@O	SER_396@HG	SER_396@OG	70,8
LYS_308@O	PHE_393@H	PHE_393@N	70,6
ASN_321@OD1	LYS_347@H	LYS_347@N	69,9
VAL_422@O	VAL_394@H	VAL_394@N	69,4

THR_453@O	GLY_423@H	GLY_423@N	69,3
GLY_403@O	ALA_407@H	ALA_407@N	68,0
GLY_552@O	THR_606@H	THR_606@N	67,3
THR_589@O	GLU_499@H	GLU_499@N	67,1
VAL_392@O	VAL_422@H	VAL_422@N	67,1
ILE_334@O	GLY_311@H	GLY_311@N	66,8
ALA_605@O	LEU_518@H	LEU_518@N	66,7
PRO_368@O	LYS_372@H	LYS_372@N	66,5
PRO_471@O	ALA_475@H	ALA_475@N	66,1
LEU_518@O	ALA_605@H	ALA_605@N	65,4
THR_606@O	GLN_551@H	GLN_551@N	64,4
ALA_411@O	LYS_415@H	LYS_415@N	64,0
SER_491@O	ALA_495@H	ALA_495@N	63,7
LYS_535@O	SER_539@HG	SER_539@OG	62,8
ILE_457@O	ARG_427@H	ARG_427@N	62,2
VAL_531@O	LYS_535@H	LYS_535@N	62,1
ASN_317@O	ASN_321@H	ASN_321@N	61,7
ARG_379@O	GLU_383@H	GLU_383@N	61,2
THR_395@O	VAL_312@H	VAL_312@N	61,0
SER_524@O	ILE_599@H	ILE_599@N	60,7
THR_602@O	ASN_556@H	ASN_556@N	60,3
GLY_355@O	THR_359@HG1	THR_359@OG1	60,2
GLU_567@OE1	ARG_529@HH21	ARG_529@NH2	60,2
ASP_480@OD2	ARG_322@HH21	ARG_322@NH2	59,1
VAL_487@O	SER_491@H	SER_491@N	58,5
ARG_436@O	ALA_440@H	ALA_440@N	58,0
LEU_483@O	VAL_487@H	VAL_487@N	57,8
GLU_581@O	VAL_583@H	VAL_583@N	57,2
VAL_444@O	LYS_448@H	LYS_448@N	57,1
ILE_336@O	ASN_317@HD21	ASN_317@ND2	56,9
LEU_565@O	GLN_569@H	GLN_569@N	56,3
GLY_515@O	LEU_454@H	LEU_454@N	56,2
ALA_533@O	ALA_537@H	ALA_537@N	56,1
GLU_567@OE2	ARG_529@HE	ARG_529@NE	55,3
ARG_529@O	ALA_533@H	ALA_533@N	54,9
LEU_462@O	VAL_466@H	VAL_466@N	54,9
ILE_310@O	THR_395@H	THR_395@N	54,9
VAL_600@O	THR_558@H	THR_558@N	54,7
VAL_309@O	ILE_334@H	ILE_334@N	54,5
ILE_510@O	GLN_514@HE22	GLN_514@NE2	54,3
GLU_474@O	GLU_478@H	GLU_478@N	53,6
ASN_528@O	GLU_532@H	GLU_532@N	53,6
VAL_409@O	ILE_413@H	ILE_413@N	53,0
ASP_597@O	ARG_529@HH11	ARG_529@NH1	53,0
ARG_484@O	GLN_488@H	GLN_488@N	52,5
LYS_412@O	GLU_416@H	GLU_416@N	52,3
PRO_408@O	LYS_412@H	LYS_412@N	52,2
ASN_481@O	GLN_485@H	GLN_485@N	51,9
GLY_355@O	THR_359@H	THR_359@N	51,9

LYS_373@O	GLU_377@H	GLU_377@N	51,2
VAL_482@O	GLY_486@H	GLY_486@N	51,2
ASN_556@O	THR_602@H	THR_602@N	51,1
THR_338@O	GLN_353@HE22	GLN_353@NE2	50,8
PHE_429@O	GLU_432@H	GLU_432@N	50,1
GLU_432@O	ARG_436@H	ARG_436@N	50,1

Table S7. Inter-molecular hydrogen bond analysis carried out on monomer 1-2 of the MD simulation of apo-FtsZ dimer. Only occurrences higher than 20% are reported. To the residue number in monomer 2, 304 units needs to be subtracted to obtain the original residue number (ASP_467 = ASP_163).

Acceptor	Donor	DonorH	%
LEU_259@O	MET_472@H	MET_472@N	80,9
GLU_195@OE1	ARG_322@HH12	ARG_322@NH1	44,1
THR_470@O	LEU_261@H	LEU_261@N	28,7
GLU_195@OE2	ARG_322@HH22	ARG_322@NH2	22,0
GLU_195@OE2	ARG_322@HH12	ARG_322@NH1	20,2

Figure S6. A) Root of mean squared deviation (RMSD), B) Root of mean squared fluctuation RMSF, C) Gyration Radius (RG) and D) Solvent Accessible Surface Area (SASA) obtained by CPPTRAJ post-processing analysis of 100ns MD simulation of FtsZ dimer in presence of GTP and Ca^{2+}. All the analysis are carried out on 5000 snapshots obtained from the simulation.

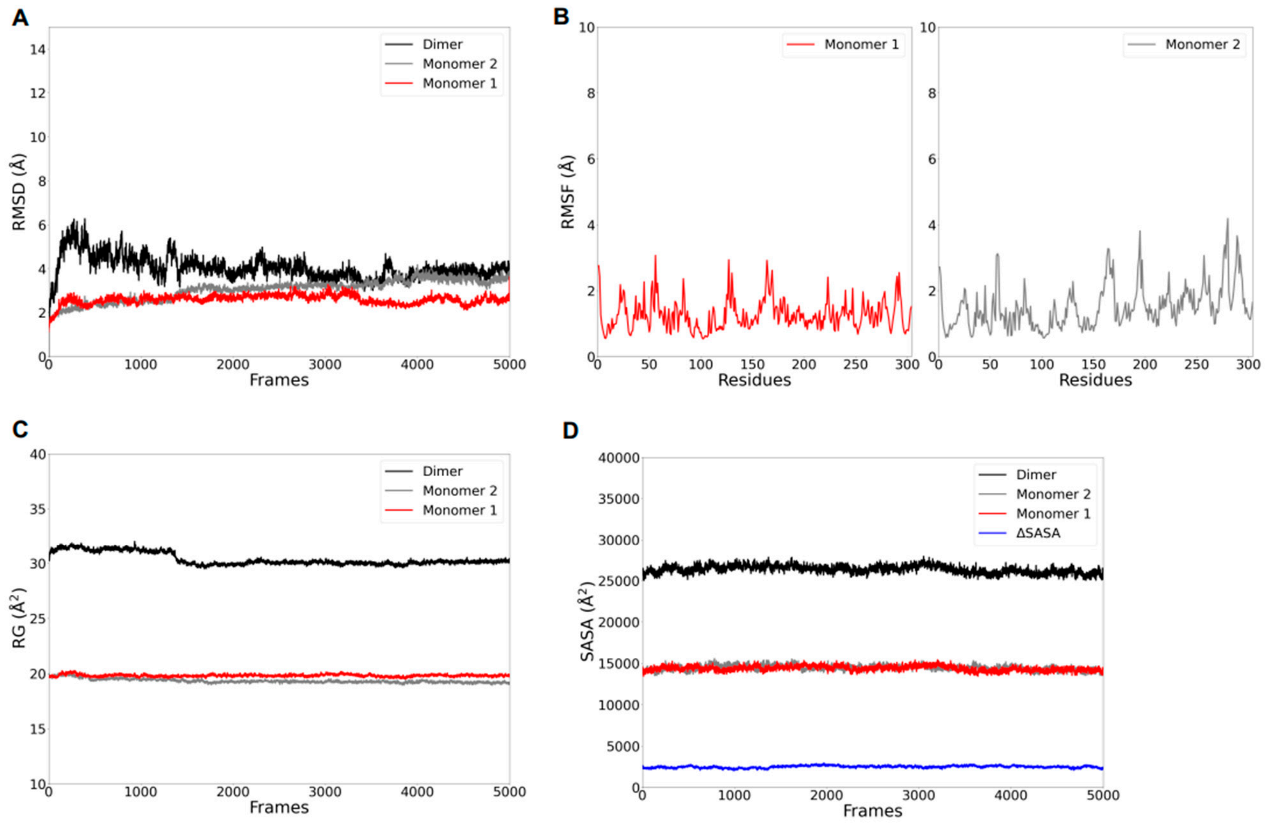

Table S8. Intra-molecular hydrogen bond analysis carried out on monomer 1 of the MD simulation of FtsZ dimer in presence of GTP and Ca^{2+}. Only occurrences higher than 50% are reported.

Acceptor	DonorH	Donor	%
ASP_176@OD2	ARG_18@HH21	ARG_18@NH2	91,4
VAL_120@O	THR_91@HG1	THR_91@OG1	89,8
ALA_201@O	THR_205@HG1	THR_205@OG1	89,6
PHE_89@O	ILE_6@H	ILE_6@N	85,1
GLY_9@O	ASN_13@H	ASN_13@N	85,0
ILE_300@O	LEU_250@H	LEU_250@N	84,9
GLU_28@O	VAL_5@H	VAL_5@N	84,5
GLY_284@O	THR_285@HG1	THR_285@OG1	84,4
VAL_296@O	THR_254@H	THR_254@N	81,5
GLY_255@O	ASN_288@H	ASN_288@N	80,6
PHE_200@O	LYS_204@H	LYS_204@N	80,1
GLU_263@OE2	ARG_225@HH21	ARG_225@NH2	78,7
THR_117@O	ASP_148@H	ASP_148@N	77,9
GLU_263@OE1	ARG_225@HE	ARG_225@NE	77,5
VAL_118@O	VAL_90@H	VAL_90@N	76,5

THR_254@O	VAL_296@H	VAL_296@N	76,3
GLY_218@O	VAL_297@H	VAL_297@N	75,0
ILE_253@O	VAL_286@H	VAL_286@N	73,6
LEU_116@O	VAL_88@H	VAL_88@N	72,4
ALA_301@O	LEU_214@H	LEU_214@N	72,4
ILE_66@O	ALA_70@H	ALA_70@N	72,2
LEU_250@O	ILE_300@H	ILE_300@N	71,6
VAL_121@O	ILE_153@H	ILE_153@N	70,6
LYS_4@O	PHE_89@H	PHE_89@N	70,1
ILE_30@O	GLY_7@H	GLY_7@N	70,0
ASP_293@O	ARG_225@HH11	ARG_225@NH1	69,6
ASN_13@OD1	ASN_33@HD22	ASN_33@ND2	69,5
ASP_163@O	THR_166@HG1	THR_166@OG1	69,0
ALA_70@O	SER_74@HG	SER_74@OG	68,0
VAL_140@O	LYS_144@H	LYS_144@N	67,6
ASN_17@OD1	LYS_43@H	LYS_43@N	67,1
PRO_64@O	LYS_68@H	LYS_68@N	67,0
VAL_8@O	SER_92@HG	SER_92@OG	66,2
ASN_252@O	THR_298@H	THR_298@N	65,2
GLY_139@O	MET_143@H	MET_143@N	64,9
ARG_180@O	GLN_184@H	GLN_184@N	64,4
VAL_5@O	ILE_30@H	ILE_30@N	64,0
ILE_6@O	THR_91@H	THR_91@N	63,2
SER_220@O	ILE_295@H	ILE_295@N	62,9
THR_98@O	ALA_102@H	ALA_102@N	62,8
THR_122@O	MET_94@H	MET_94@N	62,8
THR_91@O	VAL_8@H	VAL_8@N	62,4
ILE_32@O	GLY_9@H	GLY_9@N	62,3
VAL_297@O	GLY_218@H	GLY_218@N	61,9
VAL_227@O	LYS_231@H	LYS_231@N	61,8
VAL_88@O	VAL_118@H	VAL_118@N	61,1
ARG_130@O	THR_134@H	THR_134@N	60,9
GLU_277@O	VAL_279@H	VAL_279@N	60,0
GLY_10@O	ASN_14@H	ASN_14@N	59,5
ASP_188@OD1	THR_298@HG1	THR_298@OG1	59,5
GLY_248@O	THR_302@H	THR_302@N	59,4
LEU_214@O	ALA_301@H	ALA_301@N	59,4
GLY_119@O	ILE_151@H	ILE_151@N	59,1
ALA_115@O	THR_117@HG1	THR_117@OG1	58,6
THR_149@O	GLY_119@H	GLY_119@N	58,6
ALA_107@O	LYS_111@H	LYS_111@N	58,6
GLN_181@O	GLY_185@H	GLY_185@N	58,1
ASP_176@OD2	ARG_18@HE	ARG_18@NE	56,8
GLY_216@O	VAL_299@H	VAL_299@N	56,7
ALA_136@O	VAL_140@H	VAL_140@N	56,7
ARG_130@O	THR_134@HG1	THR_134@OG1	56,6
VAL_105@O	ILE_109@H	ILE_109@N	56,3
VAL_249@O	ILE_282@H	ILE_282@N	55,9
ALA_102@O	VAL_106@H	VAL_106@N	55,6

ARG_225@O	ALA_229@H	ALA_229@N	55,5
ASN_280@O	VAL_249@H	VAL_249@N	55,4
GLY_7@O	ILE_32@H	ILE_32@N	55,2
GLY_51@O	THR_55@H	THR_55@N	55,1
ILE_153@O	ARG_123@H	ARG_123@N	54,8
LYS_69@O	GLU_73@H	GLU_73@N	54,7
GLU_65@O	LYS_69@H	LYS_69@N	54,5
VAL_183@O	SER_187@HG	SER_187@OG	54,3
THR_302@O	GLN_247@H	GLN_247@N	53,8
VAL_178@O	GLY_182@H	GLY_182@N	53,1
PRO_104@O	LYS_108@H	LYS_108@N	52,5
GLY_211@O	LEU_150@H	LEU_150@N	52,4
VAL_90@O	VAL_120@H	VAL_120@N	52,2
LYS_68@O	GLU_72@H	GLU_72@N	52,2
ASP_148@OD2	THR_117@H	THR_117@N	52,1
LYS_204@O	SER_208@HG	SER_208@OG	51,7
LYS_108@O	GLU_112@H	GLU_112@N	51,4
ASP_176@OD1	ARG_18@HE	ARG_18@NE	51,2
ASN_224@O	GLU_228@H	GLU_228@N	51,0
ASP_176@O	ARG_180@H	ARG_180@N	50,7
ALA_275@O	GLY_245@H	GLY_245@N	50,7
ASN_33@O	ILE_50@H	ILE_50@N	50,0

Table S9. Intra-molecular hydrogen bond analysis carried out on monomer 2 of the MD simulation of FtsZ dimer in presence of GTP and Ca^{2+}. Only occurrences higher than 50% are reported. To the residue number in monomer 2, 304 units needs to be subtracted to obtain the original residue number (ASP_467 = ASP_163).

Acceptor	DonorH	Donor	%
ASP_469@O	THR_472@HG1	THR_472@OG1	95,2
VAL_592@O	THR_560@HG1	THR_560@OG1	93,8
ALA_507@O	THR_511@HG1	THR_511@OG1	85,5
GLY_561@O	ASN_594@H	ASN_594@N	81,6
ASP_482@O	ARG_486@H	ARG_486@N	81,2
GLU_334@O	VAL_311@H	VAL_311@N	81,0
VAL_427@O	ILE_459@H	ILE_459@N	79,6
ILE_606@O	LEU_556@H	LEU_556@N	79,5
ARG_436@O	THR_440@HG1	THR_440@OG1	78,3
LEU_556@O	ILE_606@H	ILE_606@N	78,3
VAL_314@O	SER_398@HG	SER_398@OG	78,3
GLY_524@O	VAL_603@H	VAL_603@N	78,0
PHE_395@O	ILE_312@H	ILE_312@N	77,7
ILE_312@O	THR_397@H	THR_397@N	77,3
THR_423@O	ASP_454@H	ASP_454@N	76,9
GLY_522@O	VAL_605@H	VAL_605@N	76,9
LEU_422@O	VAL_394@H	VAL_394@N	75,9
ILE_372@O	ALA_376@H	ALA_376@N	75,3
GLY_425@O	ILE_457@H	ILE_457@N	75,3
GLY_315@O	ASN_319@H	ASN_319@N	74,5

VAL_424@O	VAL_396@H	VAL_396@N	74,1
PHE_506@O	LYS_510@H	LYS_510@N	73,7
LYS_310@O	PHE_395@H	PHE_395@N	70,9
THR_604@O	ASN_558@H	ASN_558@N	70,7
ILE_336@O	GLY_313@H	GLY_313@N	70,5
THR_455@O	GLY_425@H	GLY_425@N	70,2
GLY_316@O	ASN_320@H	ASN_320@N	69,0
ASP_482@OD1	ARG_324@HH21	ARG_324@NH2	68,6
GLY_554@O	THR_608@H	THR_608@N	68,3
LYS_437@O	GLN_441@H	GLN_441@N	68,2
ASN_323@OD1	LYS_349@H	LYS_349@N	67,5
PRO_370@O	LYS_374@H	LYS_374@N	66,4
VAL_533@O	LYS_537@H	LYS_537@N	65,9
ARG_436@O	THR_440@H	THR_440@N	65,7
GLY_445@O	MET_449@H	MET_449@N	64,6
LYS_537@O	SER_541@HG	SER_541@OG	64,5
GLY_357@O	THR_361@HG1	THR_361@OG1	64,3
THR_560@O	VAL_602@H	VAL_602@N	64,1
VAL_394@O	VAL_424@H	VAL_424@N	63,9
ALA_376@O	SER_380@HG	SER_380@OG	63,3
GLY_517@O	LEU_456@H	LEU_456@N	63,1
ASP_482@OD2	ARG_324@HE	ARG_324@NE	63,0
VAL_489@O	SER_493@H	SER_493@N	63,0
ILE_338@O	GLY_315@H	GLY_315@N	62,9
SER_526@O	ILE_601@H	ILE_601@N	62,6
MET_557@O	GLY_590@H	GLY_590@N	62,6
LEU_520@O	ALA_607@H	ALA_607@N	61,4
ALA_413@O	LYS_417@H	LYS_417@N	61,3
ALA_421@O	THR_423@HG1	THR_423@OG1	60,4
VAL_605@O	GLY_522@H	GLY_522@N	60,3
VAL_311@O	ILE_336@H	ILE_336@N	59,3
GLY_357@O	THR_361@H	THR_361@N	59,2
THR_406@OG1	GLY_401@H	GLY_401@N	58,6
ASP_482@OD2	ARG_486@HH11	ARG_486@NH1	58,4
THR_397@O	VAL_314@H	VAL_314@N	57,8
ALA_607@O	LEU_520@H	LEU_520@N	57,2
LEU_485@O	VAL_489@H	VAL_489@N	56,8
ASP_508@O	ILE_512@H	ILE_512@N	56,3
THR_340@O	GLN_355@HE22	GLN_355@NE2	55,0
VAL_603@O	GLY_524@H	GLY_524@N	54,9
THR_404@O	ALA_408@H	ALA_408@N	54,6
LEU_567@O	GLN_571@H	GLN_571@N	54,5
LYS_374@O	GLU_378@H	GLU_378@N	54,5
GLN_487@O	GLY_491@H	GLY_491@N	54,4
ARG_438@O	ALA_442@H	ALA_442@N	53,7
VAL_322@O	ILE_326@H	ILE_326@N	53,5
ALA_408@O	VAL_412@H	VAL_412@N	53,3
ARG_381@O	GLU_385@H	GLU_385@N	53,1
LYS_414@O	GLU_418@H	GLU_418@N	52,6

ILE_338@O	ASN_319@HD21	ASN_319@ND2	52,6
GLY_405@O	ALA_409@H	ALA_409@N	52,5
ASN_530@O	GLU_534@H	GLU_534@N	52,2
GLU_569@OE1	ARG_531@HH21	ARG_531@NH2	52,1
GLN_490@O	ASP_494@H	ASP_494@N	52,1
ALA_442@O	VAL_446@H	VAL_446@N	52,0
ASP_392@O	LEU_422@H	LEU_422@N	51,4
THR_608@O	GLN_553@H	GLN_553@N	51,4
ALA_535@O	ALA_539@H	ALA_539@N	51,2
ALA_337@O	ILE_354@H	ILE_354@N	51,0
VAL_396@O	VAL_426@H	VAL_426@N	50,9
VAL_484@O	GLY_488@H	GLY_488@N	50,8
LEU_456@O	ALA_519@H	ALA_519@N	50,6
ASN_558@O	THR_604@H	THR_604@N	50,3
ASN_319@O	ASN_323@H	ASN_323@N	50,2
ILE_459@O	ARG_429@H	ARG_429@N	50,1
VAL_446@O	LYS_450@H	LYS_450@N	50,1

Table S10. Inter-molecular hydrogen bond analysis carried out on monomers 1-2 of the MD simulation of FtsZ dimer in presence of GTP and Ca^{2+}. Only occurrences higher than 20% are reported. To the residue number in monomer 2, 304 units needs to be subtracted to obtain the original residue number (ASP_467 = ASP_163).

Acceptor	DonorH	Donor	%
LEU_259@O	MET_474@H	MET_474@N	68,5
MET_281@O	ARG_436@H	ARG_436@N	39,3
ASN_197@O	ARG_438@HH21	ARG_438@NH2	23,8
ASP_202@OD2	LYS_437@HZ2	LYS_437@NZ	20,2
THR_472@O	LEU_261@H	LEU_261@N	49,2
LYS_470@O	SER_260@HG	SER_260@OG	47,6
MET_281@O	ARG_436@H	ARG_436@N	39,3
ASN_197@O	ARG_438@HH21	ARG_438@NH2	23,8
ASP_202@OD2	LYS_437@HZ2	LYS_437@NZ	20,2

Figure S7. A) Root of mean squared deviation (RMSD), B) Root of mean squared fluctuation RMSF, C) Gyration Radius (RG) and D) Solvent Accessible Surface Area (SASA) obtained by CPPTRAJ post-processing analysis of 100ns MD simulation of FtsZ dimer in presence of GTP, $Ca^{2+}$and compound **11**. All the analysis are carried out on 5000 snapshots obtained from the simulation.

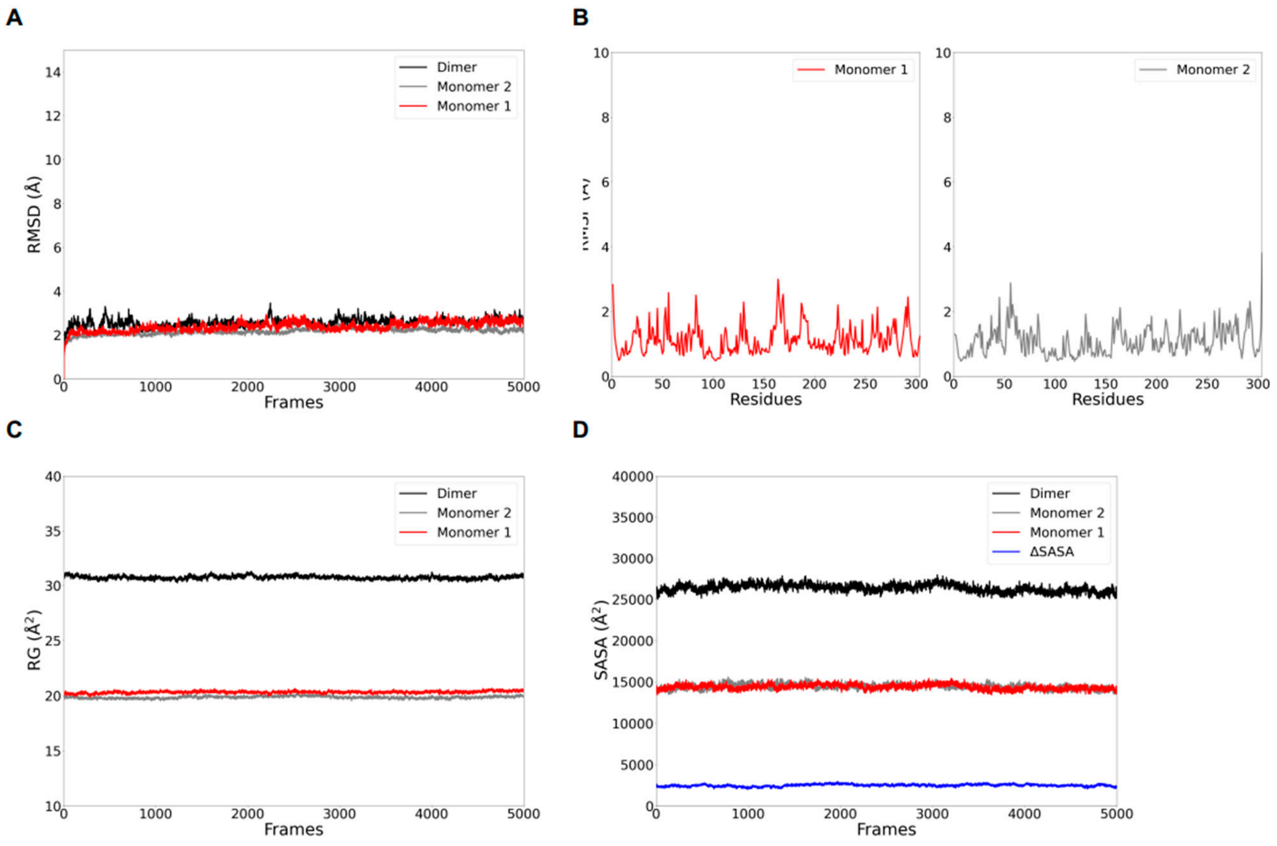

Table S11. Intra-molecular hydrogen bond analysis carried out on monomer 1 of the MD simulation of FtsZ dimer in presence of GTP, $Ca^{2+}$and compound **11**. Only occurrences higher than 50% are reported.

Acceptor	DonorH	Donor	%
ASP_176@OD2	ARG_18@HH21	ARG_18@NH2	96,4
ALA_201@O	THR_205@HG1	THR_205@OG1	91,9
VAL_120@O	THR_91@HG1	THR_91@OG1	90,8
LEU_58@O	THR_34@HG1	THR_34@OG1	89,4
LEU_250@O	ILE_300@H	ILE_300@N	87,3
ILE_300@O	LEU_250@H	LEU_250@N	83,5
VAL_296@O	THR_254@H	THR_254@N	83,3
PHE_89@O	ILE_6@H	ILE_6@N	83,2
GLY_255@O	ASN_288@H	ASN_288@N	79,9
THR_117@O	ASP_148@H	ASP_148@N	78,8
GLU_28@O	VAL_5@H	VAL_5@N	76,0
ALA_301@O	LEU_214@H	LEU_214@N	76,0
THR_254@O	VAL_296@H	VAL_296@N	75,1

LEU_116@O	VAL_88@H	VAL_88@N	74,8
ILE_66@O	ALA_70@H	ALA_70@N	73,1
ALA_175@O	LEU_179@H	LEU_179@N	71,6
ASP_163@O	THR_166@HG1	THR_166@OG1	71,4
ASP_293@O	ARG_225@HH11	ARG_225@NH1	70,7
GLY_218@O	VAL_297@H	VAL_297@N	70,2
GLY_9@O	ASN_13@H	ASN_13@N	69,5
ILE_6@O	THR_91@H	THR_91@N	69,5
ILE_253@O	VAL_286@H	VAL_286@N	68,9
VAL_118@O	VAL_90@H	VAL_90@N	68,4
ILE_30@O	GLY_7@H	GLY_7@N	68,2
GLY_119@O	ILE_151@H	ILE_151@N	68,0
VAL_140@O	LYS_144@H	LYS_144@N	67,4
PRO_64@O	LYS_68@H	LYS_68@N	67,4
ASN_17@OD1	LYS_43@H	LYS_43@N	66,8
ILE_32@O	GLY_9@H	GLY_9@N	66,5
ASP_148@O	GLY_211@H	GLY_211@N	66,3
ILE_153@O	ARG_123@H	ARG_123@N	66,1
GLY_139@O	MET_143@H	MET_143@N	66,0
VAL_105@O	ILE_109@H	ILE_109@N	65,9
ALA_70@O	SER_74@HG	SER_74@OG	65,6
THR_149@O	GLY_119@H	GLY_119@N	65,0
THR_100@OG1	GLY_95@H	GLY_95@N	64,0
GLY_284@O	THR_285@HG1	THR_285@OG1	63,8
PHE_200@O	LYS_204@H	LYS_204@N	63,7
ARG_130@O	THR_134@H	THR_134@N	63,2
VAL_227@O	LYS_231@H	LYS_231@N	62,3
GLY_10@O	ASN_14@H	ASN_14@N	62,2
ILE_282@O	MET_251@H	MET_251@N	61,9
ALA_115@O	THR_117@HG1	THR_117@OG1	61,5
THR_302@O	GLN_247@H	GLN_247@N	61,3
ASP_176@OD1	ARG_18@HE	ARG_18@NE	61,2
ARG_75@O	GLU_79@H	GLU_79@N	60,9
VAL_5@O	ILE_30@H	ILE_30@N	60,6
VAL_121@O	ILE_153@H	ILE_153@N	60,2
LYS_4@O	PHE_89@H	PHE_89@N	60,0
ASN_224@O	GLU_228@H	GLU_228@N	59,7
ALA_136@O	VAL_140@H	VAL_140@N	59,7
VAL_8@O	SER_92@HG	SER_92@OG	59,3
VAL_88@O	VAL_118@H	VAL_118@N	59,0
ALA_107@O	LYS_111@H	LYS_111@N	58,5
VAL_90@O	VAL_120@H	VAL_120@N	57,7
ALA_31@O	ILE_48@H	ILE_48@N	57,3
ARG_180@O	GLN_184@H	GLN_184@N	56,7
GLY_96@O	GLN_135@HE22	GLN_135@NE2	56,3
ALA_102@O	VAL_106@H	VAL_106@N	55,5
THR_34@O	GLN_49@HE22	GLN_49@NE2	55,2
SER_220@O	ILE_295@H	ILE_295@N	54,8
ASN_13@O	ASN_17@H	ASN_17@N	54,7

VAL_297@O	GLY_218@H	GLY_218@N	54,7
GLY_248@O	THR_302@H	THR_302@N	54,7
GLU_277@O	VAL_279@H	VAL_279@N	54,5
THR_91@O	VAL_8@H	VAL_8@N	53,9
LYS_69@O	GLU_73@H	GLU_73@N	53,3
VAL_249@O	ILE_282@H	ILE_282@N	53,2
GLY_99@O	ALA_103@H	ALA_103@N	53,0
LYS_108@O	GLU_112@H	GLU_112@N	52,8
GLU_65@O	LYS_69@H	LYS_69@N	51,9
GLN_184@OE1	ARG_180@HE	ARG_180@NE	51,3
GLN_133@O	ALA_137@H	ALA_137@N	51,1
THR_298@O	ASN_252@H	ASN_252@N	51,1
LEU_3@O	GLU_28@H	GLU_28@N	50,8

Table S12. Intra-molecular hydrogen bond analysis carried out on monomer 2 of the MD simulation of FtsZ dimer in presence of GTP, $Ca^{2+}$and compound **11**. Only occurrences higher than 50% are reported. To the residue number in monomer 2, 304 units needs to be subtracted to obtain the original residue number (ASP_467 = ASP_163).

Acceptor	DonorH	Donor	%
ASP_482@OD2	ARG_324@HH21	ARG_324@NH2	99,2
VAL_314@O	SER_398@HG	SER_398@OG	95,8
VAL_426@O	THR_397@HG1	THR_397@OG1	92,8
GLU_434@OE1	ARG_438@HH11	ARG_438@NH1	92,2
ALA_507@O	THR_511@HG1	THR_511@OG1	89,4
ILE_606@O	LEU_556@H	LEU_556@N	87,9
GLY_425@O	ILE_457@H	ILE_457@N	87,4
ILE_336@O	GLY_313@H	GLY_313@N	86,4
THR_397@O	VAL_314@H	VAL_314@N	86,2
ASP_482@O	ARG_486@H	ARG_486@N	85,7
THR_423@O	ASP_454@H	ASP_454@N	84,3
GLY_561@O	ASN_594@H	ASN_594@N	84,1
ILE_372@O	ALA_376@H	ALA_376@N	83,4
VAL_427@O	ILE_459@H	ILE_459@N	82,8
VAL_602@O	THR_560@H	THR_560@N	81,0
LEU_422@O	VAL_394@H	VAL_394@N	80,1
ARG_486@O	GLN_490@H	GLN_490@N	80,1
PHE_395@O	ILE_312@H	ILE_312@N	79,5
ILE_459@O	ARG_429@H	ARG_429@N	79,5
VAL_424@O	VAL_396@H	VAL_396@N	79,3
ALA_607@O	LEU_520@H	LEU_520@N	79,1
GLY_524@O	VAL_603@H	VAL_603@N	78,6
ALA_376@O	SER_380@HG	SER_380@OG	75,3
ALA_442@O	VAL_446@H	VAL_446@N	74,6
ASP_482@OD1	ARG_324@HE	ARG_324@NE	73,4
ARG_436@O	THR_440@HG1	THR_440@OG1	73,3
GLY_445@O	MET_449@H	MET_449@N	73,1

ILE_312@O	THR_397@H	THR_397@N	71,5
THR_560@O	VAL_602@H	VAL_602@N	71,0
GLU_334@O	VAL_311@H	VAL_311@N	70,9
SER_398@O	THR_428@H	THR_428@N	70,6
ASP_469@O	THR_472@HG1	THR_472@OG1	69,3
PHE_506@O	LYS_510@H	LYS_510@N	69,1
ALA_421@O	THR_423@HG1	THR_423@OG1	68,8
ILE_559@O	VAL_592@H	VAL_592@N	68,8
ALA_321@O	MET_325@H	MET_325@N	67,7
ARG_381@O	GLU_385@H	GLU_385@N	67,5
ARG_436@O	THR_440@H	THR_440@N	67,1
LEU_556@O	ILE_606@H	ILE_606@N	66,6
GLY_357@O	THR_361@H	THR_361@N	66,3
GLY_317@O	ALA_321@H	ALA_321@N	65,8
THR_404@O	ALA_408@H	ALA_408@N	64,7
THR_404@OG1	THR_340@HG1	THR_340@OG1	64,6
ILE_338@O	ASN_319@HD21	ASN_319@ND2	64,2
THR_608@O	GLN_553@H	GLN_553@N	64,2
ASN_323@OD1	LYS_349@H	LYS_349@N	63,7
SER_526@O	ILE_601@H	ILE_601@N	62,9
MET_400@O	THR_406@HG1	THR_406@OG1	62,8
VAL_502@O	THR_591@H	THR_591@N	62,6
ASP_508@OD2	ASP_505@H	ASP_505@N	62,3
GLY_590@O	THR_591@HG1	THR_591@OG1	62,2
ILE_588@O	MET_557@H	MET_557@N	62,2
VAL_555@O	ILE_588@H	ILE_588@N	61,6
ASN_558@O	THR_604@H	THR_604@N	60,9
THR_455@O	GLY_425@H	GLY_425@N	60,6
VAL_603@O	GLY_524@H	GLY_524@N	60,6
VAL_396@O	VAL_426@H	VAL_426@N	60,3
GLY_522@O	VAL_605@H	VAL_605@N	60,1
VAL_446@O	LYS_450@H	LYS_450@N	60,1
VAL_489@O	SER_493@HG	SER_493@OG	59,9
ASP_392@O	LEU_422@H	LEU_422@N	58,7
VAL_411@O	ILE_415@H	ILE_415@N	58,3
GLU_583@O	VAL_585@H	VAL_585@N	57,4
LEU_485@O	VAL_489@H	VAL_489@N	57,0
ASN_530@O	GLU_534@H	GLU_534@N	56,9
ARG_438@O	ALA_442@H	ALA_442@N	56,6
LEU_520@O	ALA_607@H	ALA_607@N	56,0
ASP_469@O	THR_472@H	THR_472@N	55,3
VAL_509@O	MET_513@H	MET_513@N	55,3
ALA_408@O	VAL_412@H	VAL_412@N	55,1
VAL_533@O	LYS_537@H	LYS_537@N	54,9
ALA_413@O	LYS_417@H	LYS_417@N	54,8
VAL_394@O	VAL_424@H	VAL_424@N	54,8
LYS_375@O	GLU_379@H	GLU_379@N	54,8
ASP_599@O	ARG_531@HH11	ARG_531@NH1	54,3
LYS_310@O	PHE_395@H	PHE_395@N	53,9

GLU_569@OE1	ARG_531@HH21	ARG_531@NH2	53,7
LYS_437@O	GLN_441@H	GLN_441@N	52,9
LEU_567@O	GLN_571@H	GLN_571@N	51,9
VAL_311@O	ILE_336@H	ILE_336@N	51,9
ILE_415@O	MET_419@H	MET_419@N	51,1
ASN_319@O	ASN_323@H	ASN_323@N	50,7
ALA_581@O	GLY_551@H	GLY_551@N	50,6
GLY_313@O	ILE_338@H	ILE_338@N	50,3
THR_428@O	MET_400@H	MET_400@N	50,0

Table S13. Inter-molecular hydrogen bond analysis carried out on monomer 1-2 of the MD simulation of FtsZ dimer in presence of GTP, Ca^{2+} and compound **11**. Only occurrences higher than 20% are reported. To the residue number in monomer 2, 304 units needs to be subtracted to obtain the original residue number (ASP_467 = ASP_163)

Acceptor	DonorH	Donor	%
LEU_259@O	MET_474@H	MET_474@N	86,3
LYS_470@O	SER_260@HG	SER_260@OG	41,0
GTP_612@O5	ASN_197@H	ASN_197@N	30,3
GTP_612@O6	ASN_197@H	ASN_197@N	28,9
GLN_265@OE1	SER_432@HG	SER_432@OG	27,3
GTP_612@O3	ASN_197@HD22	ASN_197@ND2	27,0
